# Megacity precipitationsheds reveal tele-connected water security challenges

**DOI:** 10.1371/journal.pone.0194311

**Published:** 2018-03-13

**Authors:** Patrick W. Keys, Lan Wang-Erlandsson, Line J. Gordon

**Affiliations:** 1 School of Global Environmental Sustainability, Colorado State University, Fort Collins, United States of America; 2 Stockholm Resilience Centre, Stockholm University, Stockholm, Sweden; 3 Department of Water Management, Faculty of Civil Engineering and Geosciences, Delft University of Technology, Delft, The Netherlands; 4 Research Institute for Humanity and Nature, Kyoto, Japan; Uppsala Universitet Teknisk-naturvetenskapliga fakulteten, SWEDEN

## Abstract

Urbanization is a global process that has taken billions of people from the rural countryside to concentrated urban centers, adding pressure to existing water resources. Many cities are specifically reliant on renewable freshwater regularly refilled by precipitation, rather than fossil groundwater or desalination. A precipitationshed can be considered the “watershed of the sky” and identifies the origin of precipitation falling in a given region. In this paper, we use this concept to determine the sources of precipitation that supply renewable water in the watersheds of the largest cities of the world. We quantify the sources of precipitation for 29 megacities and analyze their differences between dry and wet years. Our results reveal that 19 of 29 megacities depend for more than a third of their water supply on evaporation from land. We also show that for many of the megacities, the terrestrial dependence is higher in dry years. This high dependence on terrestrial evaporation for their precipitation exposes these cities to potential land-use change that could reduce the evaporation that generates precipitation. Combining indicators of water stress, moisture recycling exposure, economic capacity, vegetation-regulated evaporation, land-use change, and dry-season moisture recycling sensitivity reveals four highly vulnerable megacities (Karachi, Shanghai, Wuhan, and Chongqing). A further six megacities were found to have medium vulnerability with regard to their water supply. We conclude that understanding how upwind landscapes affect downwind municipal water resources could be a key component for understanding the complexity of urban water security.

## Introduction

Urban environments are home to the most rapidly growing human populations on the planet [1]. Water is just as important in cities as it is elsewhere, but the concentration of people within cities makes the sustainable supply of clean freshwater a particularly urgent priority for science and policy [2]. Globally, cities rely on a wide range of water sources, including groundwater, lakes, reservoirs, and in some cases desalinated ocean water [3]. Research on urban water supplies often focuses on how surface and groundwater can be managed. However, water supply is also affected by precipitation systems including the evaporation from land uses far away from cities [4]. This connection in the atmospheric water cycle, where evaporation from one location travels through the atmosphere to fall as precipitation in another location, is called moisture recycling.

Urban water only comes from a few different types of sources. Surface water generally comes from either direct rain runoff, or from snow or glacial melt, and is overwhelmingly the most common source of water for urban areas globally [3]. Groundwater can come either in the form of fossil aquifers or actively recharged aquifers [5]. Desalination, though not as widely used as surface or groundwater, is concentrated in arid areas, particularly in the oil-exporting nations of the Arabian Gulf [6]. The conventional spatial unit for surface water is the watershed, and a watershed is delineated by topography, with water flowing downhill to the lowest point, most commonly the ocean [7]. Likewise, the surface watershed is also an appropriate boundary for considering the water that is actively recharging groundwater aquifers that are near the surface [8].

The water that forms this surface runoff in the watershed originates as precipitation (i.e., rain, drizzle, snow, sleet, graupel, or hail). Previous work enables the analysis of the sources of the precipitation that falls in a given location, including in a watershed [9, 10]. Advances in global modeling enable the tracking of atmospheric moisture flowing around the planet, and furthermore can identify the specific locations where moisture enters the atmosphere as evaporation, and where it falls out as precipitation. In many parts of the world, evaporation from land later returns to land as precipitation. On average, 40% of precipitation on land originates from evaporation that came from land, but this can be substantially higher in some regions and during certain seasons [11].

**Fig 1:**
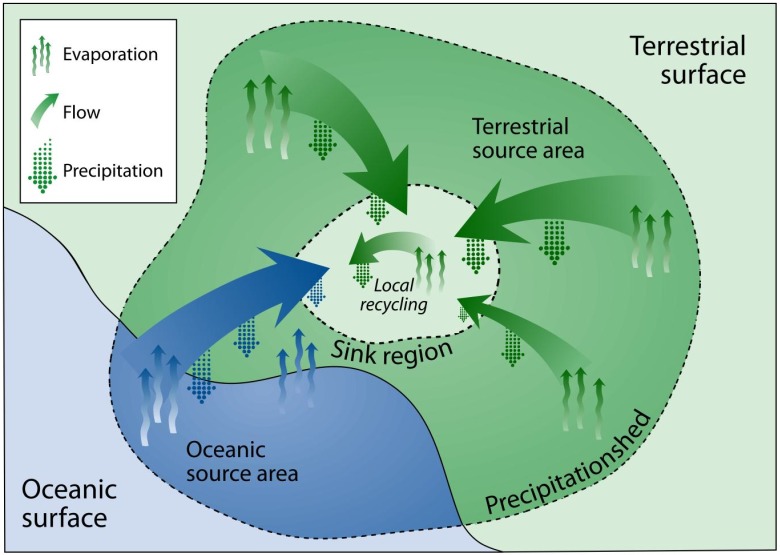
Conceptual diagram of a precipitationshed. Originally published in Keys et al. [12] and reproduced here under the Creative Commons Attribution 3.0 License.

In an effort to link the management of surface water with the sources of precipitation for a given location (e.g. a watershed), Keys et al. [12] introduced the concept of the precipitationshed, which defines a spatial boundary enclosing upwind evaporative sources of downwind precipitation (Fig 1). In the precipitationshed, a precipitation ‘sink region’ receives precipitation from upwind ‘source regions’, as well as from within the sink itself (Fig 1). Other work has found that precipitationsheds are spatially consistent among years, though the magnitude of contribution from different parts of the precipitationshed does fluctuate [13].

Importantly, anthropogenic land-use change can significantly alter terrestrial moisture recycling (Table 1; [14–23]). This potential impact reveals that tele-connections exist between distant human activities, such as large-scale forest clearing for crop production, and the amount of precipitation falling downwind in a city’s watershed [24]. The impacts to precipitation from land-use change are summarized in Table 1, with an estimate of the change in precipitation that can result from land-use changes. The significant impacts of land-use change on downwind precipitation are evident, but the impacts are different depending on geographic location (due to climatic differences and wind directions) as well as the type of land-use change. Additionally, significant differences in moisture recycling between dry and wet years have been previously explored [25, 26].

**Table 1:** Summary of literature values for land-use changes and the associated impact to downwind precipitation, listed in order of publication year [14–23].

AUTHOR	REGION	LAND USE CHANGE (LUC)	TYPE OF LUC	CHANGE IN PRECIPITATION	
				absolute	%
Bagley et al. (2012)	East Asia	Difference betweennatural vegetationand bare soil	Theoretical	- - -	-11.89%
	Cent. Asia			- - -	-16.70%
	N. America			- - -	-8.34%
	S. America			- - -	-16.90%
	W. Africa			- - -	-9.64%
Salih et al. (2013)	Sudan	Deforestation, replaced by grassor desert	Theoretical	grass: -1mm/day to +0.5 mm/day	grass: -25% to +5%
				desert: -2.1 mm/day (or more) to +0.5 mm/day	desert: -52.5% to +5%
Lo and Famiglietti (2013)	California	Irrigation replacing grassland	Observed	from: +2mm/month to 12mm/month	about +15%
Wei et al. (2013)	India	Irrigation replacing variety of different land-uses	Observed	from 120mm/yr to 10mm/yr	from +22% to +2%
	China			from 2 mm/yr to 28 mm/yr	from +0.4% to +5%
	USA			from 0.4 mm/yr to 5 mm/yr	from +0.1% to +1.1%
	Sahel			from 0.4 mm/yr to 4.5 mm/yr	from +0.2% to +3%
Tuinenburg et al. (2014)	India	Irrigation	Observed	from: -200 mm/yr in E. India to +200 in W. India, N. India, & Pakistan	from: -15% in E. India to +15 to 30% in W. India, N. India, & Pakistan
Spracklen et al. (2015)	Amazon	Deforestation (replaced by variety of land-uses depending on simulation)	Observed and Theoretical	- - -	from: -0% (with 0% deforestation) to ∼-20% (with 100% deforestation)
Swann et al. (2015)	Amazon	Deforestation (replaced bycropland)	Theoretical	from -3mm/day to +1mm/day (or -900mm/yr to +365mm/yr; but, most changes = 0)	from -25% to +17% (but, most changes = 0%)
Badger and Dirmeyer (2015)	Amazon	Deforestation (replaced by heterogenous cropland)	Theoretical	from -8mm/day to -2mm/day in NW Amazon (-1460 mm/yr to -365 mm/yr);	- - -
				from -1 mm/day to +1 mm/day in S & E Amazon (-365 mm/yr to +365mm/yr)	
Halder et al. (2016)	South Asia	Deforestation (replaced by cropland)	Observed	from -16 mm/yr to +16mm/yr	- - -
Keys et al. (2016)	Amazon	Deforestation (replaced bydesert)	Theoretical	from: -80mm/yr to -10mm/yr	from: -6% to -1%

Previous work has explored the dire situation of urban water security [3]. Likewise, other work has examined moisture recycling dynamics and vulnerability with regard to various sectors of society [12, 14], whether terrestrial moisture recycling can modulate the water cycle during droughts and dry years [25, 27], and how land-use change can modify moisture recycling (Table 1). However, there remains a gap in linking the contemporary understanding of moisture recycling with the vulnerability of urban water resources. Our aim is to contribute toward closing this research gap.

In this paper, we focus on the largest cities globally, so-called megacities that exceed 10 million people, and investigate the extent to which their water supplies are reliant on terrestrial moisture recycling, versus oceanic moisture recycling. We use megacities in this research to include (a) geographically diverse urban areas, (b) a significant fraction of the global human population, and to capture (c) both low- and high-income countries. Our key research questions are:

1. Where are the evaporation sources providing precipitation for megacity watersheds?
2. How important for megacity water supplies is terrestrial moisture recycling during dry years compared to wet years?
3. How does considering terrestrial moisture recycling modify the assessment of megacity water supply vulnerability?

For our first research question, we will identify the sources of water for each megacity in our study, and then we will identify the precipitationshed that corresponds to those water supplies. From this we will calculate how much of the moisture recycling is from land versus oceans. For our second research question, we will identify relatively dry, neutral, and wet years for each megacity by splitting the entire time period of analysis into thirds—a third with the highest average precipitation (e.g. wet years), a third with intermediate precipitation (e.g. neutral years), and a third with the lowest precipitation (e.g. dry years). Using these years, we will identify the precipitationsheds that correspond to each of these ranges of years, and identify whether the moisture recycling patterns for dry years are significantly different from the wet years. Finally, for our third research question, we will combine the output of this analysis with other water stress, land-use change, and demographic data for the megacities, to determine the vulnerability of each megacity’s moisture recycling. Each of these methods are explained in detail in the following section.

## Materials and methods

### Megacity watershed identification

McDonald et al. [3] evaluated the sources of municipal water for 632 cities globally (representing 1.7 billion people), identifying which cities were dependent on specific types of supply. They identified the geographic coordinates of all points of supply for each city, and whether they were groundwater wells, lakes, diversions from other basins, or desalination plants. As the starting point of our analysis, we used data on the population, type of water dependence, and locations of water sources from [3]. We ordered the cities from largest population to smallest population (using populations for 2015), and we identified all megacities globally. Then, we identified the spatial footprint of all water supplies including rivers, engineered diversions, reservoirs, and lakes (see [3] for details on their analysis). We also queried a database of global watersheds [28].

### Calculating the precipitationshed

To calculate the precipitationshed for each megacity we used a moisture tracking model. Each of the megacities’ watersheds was used as the sink of precipitation for the moisture recycling analysis. For example, the watershed for Cairo is the entire Nile River basin. Thus, we would use the entire Nile watershed as the sink region of precipitation, and backtrack this precipitation through the atmosphere to its origin as evaporation somewhere else on the planet.

There is a growing diversity of atmospheric moisture tracking methods and models [9, 17, 29, 30], each with various advantages and disadvantages. We use the Water Accounting Model 2layers (hereafter, WAM-2layers), which efficiently computes spatial sources of evaporation for a precipitationshed based on climate data [11]. The WAM-2layers vertically integrates water in the atmosphere into an upper and lower layer, which allows for differing wind speeds between the upper and lower atmosphere [23, 31]. Most useful for our analysis, the WAM-2layers is able to track moisture backwards in time, from where it falls as precipitation, back through the atmosphere, to the specific upwind locations where it entered the atmosphere as evaporation (for the backtracking description, see [12]).

Thus, the WAM-2layers reveals the origin of where evaporation contributes to a specific location’s precipitation, i.e. the precipitation sink. Many parts of the world contribute very small amounts of evaporation to a specific location’s precipitation, so to make the data practical, we limit the source region that we consider to all locations that contribute a minimum of 1 mm/yr of evaporation to annual sink precipitation, during every year of the analysis. This is the *core precipitationshed* (see [13]). We select 1mm since it is a common depth of precipitation that is measured on precipitation gauges, making it a reasonable lower limit for depicting moisture recycling patterns. The fraction of a sink region’s precipitation that is contained within the core precipitationshed can vary significantly, depending on the sink region, the location on the planet, and other factors. However, rather than use the boundary that contains a specific percent of a sink region’s precipitation origins (e.g. 50%), we specify a minimum depth of contribution which is easier to relate upwind changes in evaporation to changes to the land-surface.

The data we use for the WAM-2layers is the ERA-Interim reanalysis from the European Center for Mesoscale Weather Forecasting [32]. Reanalysis data are a combination of realistic weather forecasting model output, combined and updated with observations from satellites, weather balloons, radar, and surface observations. The ERA-Interim data cover the entire planet, and we downloaded the data at a gridded resolution of 1.5-degrees by 1.5-degrees, for the period 1979 to 2014. We use data at the 6- and 3-hourly timestep, including: 6-hourly specific humidity, 6-hourly winds (horizontal and vertical), 6-hourly surface pressure, and 3-hourly evaporation and precipitation. Since the winds in the atmosphere can move very fast, we must run our model at a time resolution that is able to capture water vapor flowing into and out of each tracked cell without missing water that passes through too quickly. Thus, we run the model at the 15-minute timestep, which requires interpolating the data from either 6- or 3-hourly to 15-minutes.

The results of our WAM-2layers analysis are 36-years of the origins of precipitation, for each megacity.

### Detecting the difference between dry- and wet year moisture recycling

We further analyzed the difference between dry- and wet year moisture recycling to examine whether megacities are more dependent on terrestrial moisture recycling during dry years, compared to wet years. To do this, we first calculated the total annual rainfall in each of the sink regions, for the 36 years of analysis. Second, we split this time-series of annual rainfall into dry, neutral and wet years, with 12 years in each category. We did this by finding the mean annual precipitation for each of the megacity watersheds, and subtracting this from the annual values. In this way, precipitation anomalies for each megacity watershed were calculated as,

$$P_{A}=Py-\bar{P}$$ (1)

where, *P*_*y*_ is the precipitation for current year, $\bar{P}$ is the mean precipitation for all years, and *P*_*A*_ is the anomalous precipitation for that year. We then split this time series of anomalies into thirds, with the bottom third representing ‘dry years’, the middle third representing ‘neutral years’, and the top third representing ‘wet years’. Using the dry and wet years, we identified the mean terrestrial moisture recycling ratio at the monthly time scale. Finally, we performed a two-sided student’s t-test, to determine whether the terrestrial moisture recycling ratios in the dry years were different from the wet years, using the 90% confidence interval.

We also calculated the difference in evaporation contribution during dry and wet years, and weighted each gridcell by its importance to megacity watershed rainfall. For every location in the precipitationshed (and for the dry, neutral, and wet years), we divided the evaporation contribution by total precipitation falling in the sink region. Formally,

$$\begin{array}{c} E_{i}^{\prime }=\frac{E_{i}}{P_{sink}} \end{array}$$ (2)

where, *E*_*i*_ is the annual average evaporation at location *i*, *P*_*sink*_ is the annual average precipitation falling in the sink region, and $E_{i}^{\prime }$ is the weighted evaporation contribution from location *i*. In this way, the evaporation was weighted by its importance to sink region precipitation. We then found the fractional difference between dry year and wet year evaporation contribution throughout each precipitationshed by calculating,

$$\begin{array}{c} E_{diff}^{\prime }=\frac{\left(E_{i,wet}^{\prime }-E_{i,dry}^{\prime }\right)}{E_{wet}^{\prime }} \end{array}$$ (3)

where, $E_{i,wet}^{\prime }$ is the weighted evaporation contribution from location *i* during wet years, $E_{i,dry}^{\prime }$ is the weighted evaporation contribution from location *i* during dry years, and $E_{diff}^{\prime }$ is the fractional difference in weighted evaporation contribution between wet and dry years. We plotted these results on regional maps, which are presented in the Results section.

### Megacity water security literature review

We performed a rigorous literature review for all 29 megacities regarding present and future challenges related to water resources, how moisture recycling interacts with these water resources, and whether megacity water security is highly exposed to moisture recycling dynamics.

Furthermore, we integrate the literature review of the overall moisture recycling and water security dynamics for each city, and develop an ‘exposure’ score that captures how moisture recycling dynamics are (or are not) buffered by each megacity. We use this score to weight the importance of the moisture recycling variables, so that the final vulnerability accurately reflects both the megacity and its coupling to moisture recycling dynamics. Three scores are possible: low exposure at 0.33; moderate exposure at 0.66, and high exposure at 1.0. We do not use zero, which would imply no exposure, since moisture recycling is linked in some way to all of the megacities at the very least via the atmospheric branch of the water cycle.

### Moisture recycling vulnerability analysis

The vulnerability analysis combined six different indicators, all related to threats to water supplies for the megacities: water stress (*WS*), economic capacity (*EC*), vegetation-regulated evaporation (*V*_*E*_), land-use change (*LUC*), dry-season sensitivity (*DRY*), and moisture recycling exposure (*MRE*). From the perspective of the megacity itself, it is important to understand how the different elements of this vulnerability analysis connect to one another. First, the two indicators *WS* and *EC* refer to the *a priori* vulnerability of a city’s water resources. Second, if a city receives a significant amount of precipitation from upwind vegetation (i.e. *V*_*E*_), and this vegetation is changing (i.e. *LUC*), then it’s important to understand that a city’s water resources may have the potential for further stress. Third, if the city is more reliant on terrestrial sources of rainfall during dry years (i.e. *DRY*) there is even more potential for water resources vulnerability, because during dry years available water supplies tend to be lower, while water demand is higher for nearly every type of user. Finally, the previous moisture recycling indicators are only relevant to a megacity if they can actually impact urban water stress. Thus, moisture recycling exposure (*MRE*) weights the scores of the moisture recycling indicators (i.e. *V*_*E*_, *LUC*, and *DRY*).

1. *Water stress* (*WS*) was adapted from [3], and was assigned a value of 0 for not stressed, and 2 or 3 depending on the number of different ways that water stress was identified (i.e. there were two surface water stress models, WaterGAP and Water Balance, and a detailed groundwater evaluation). The numbers start at 2 (rather than 1), to emphasize the importance of pre-existing water stress relative to the other components of vulnerability.
2. *Economic capacity* (*EC*) was adapted from [3], and was the income category of each sink region, with “Low income”, “Middle lower”, “Middle upper”, and “High income” being assigned values of 3, 2, 1, and 0 (respectively), with 3 equating to the lowest economic capacity, and 0 referring to the highest economic capacity.
3. *Vegetation-regulated evaporation* (*V*_*E*_) was taken from [23], and refers to the importance of current vegetation to regulating evaporation that returns as precipitation on land. *V*_*E*_ can range from 0% (no vegetation-regulation of evaporation) to 100% (full vegetation-regulation of evaporation). We assigned *V*_*E*_ values of <5% and <10% scores of 1 and 2 (respectively).
4. *Land-use change* (*LUC*) was calculated in this paper, and reflects the annual change in cropland cover throughout the precipitationshed. We used the HYDE dataset [33], and calculated the difference between 1990 and 2005 (with 2005 being the most recent year available, and the difference between 1990 and 2005 assumed to reflect contemporary rates of land-use change). Note, this calculation is an indicator of how land-use has changed, and does not pertain directly to evaporation, but rather represents a proxy for potential changes to evaporation as a result of changing land-use. The changes are relatively small, and represent cropland contraction (i.e. negative values) and cropland expansion (i.e. positive values). We categorized *LUC* rates of <- 0.1%/yr, -0.1%/yr < *LUC* <+0.1%/yr, and >+0.1%/yr, with the vulnerability index of 2,1,2 (respectively). Note, that we categorize both cropland expansion and contraction as more substantial land-use change, whereas any rate between -0.1%/yr and +0.1%/yr is considered as more minor land-use change. Note, that any value of 0%/yr would correspond to a value of 0 on the *LUC* indicator, but there were no such values.
5. *Dry-season sensitivity* (*DRY*) was calculated in this paper, and is defined as having two or more months that are significantly more (based on student’s t-test) reliant on terrestrial moisture recycling compared to wet years. We categorized these values with 0 as insensitive, and 3 as sensitive. We used 3 instead of 1 to highlight the important role that dry years can have on moisture recycling dynamics.
6. *Moisture recycling exposure* (*MRE*) was calculated in this paper, and is meant to characterize the exposure of urban water stress to changes in moisture recycling. Based on the literature review, and the moisture recycling information contained within this analysis, we assign an exposure score of ‘weak’, moderate’, or ‘strong’, corresponding to scores of $\frac{1}{3}$, $\frac{2}{3}$, and 1. This exposure score is multiplied by the moisture recycling indicators, producing a set of weighted moisture recycling indicators.

The vulnerability score is calculated as follows,

$$\begin{array}{c} V_{score}=\frac{WS+EC+MRE(\left(V_{E}*LUC\right)+DRY)}{V_{maximum}} \end{array}$$ (4)

where, *WS* is water stress, *EC* is economic capacity, *MRE* is moisture recycling exposure, *V*_*E*_ is vegetation regulated evaporation, *LUC* is land-use change, *DRY* is the dry-season sensitivity, and *V*_*maximum*_ is the maximum possible vulnerability, which serves to normalize the summed scores. Thus, the resulting scores range from 0 to 1. The vulnerability of each megacity’s moisture recycling was then assigned using the following,

$$\begin{array}{c} If\;V_{score}=\{\begin{array}{cc} \leq 0.2 & \;then,\;V_{score}=very\;low\\ >0.2\;and\;\leq 0.4 & \;then,\;V_{score}=low\\ >0.4\;and\;\leq 0.6 & \;then,\;V_{score}=medium\\ >0.6\;and\;\leq 0.8 & \;then,\;V_{score}=high\\ >0.8 & \;then,\;V_{score}=very\;high \end{array} \end{array}$$ (5)

where, *V*_*score*_ is the total vulnerability score for each individual megacity.

Thus, we combine analyses from previous work (i.e. *WS*, *EC*, and *V*_*E*_) with new analysis (i.e. *LUC*, *DRY*, and *MRE*) to create an integrated index of moisture recycling vulnerability. Ultimately, we recognize that we are simplifying the relationship among these different indicators, but we view this as an initial step in characterizing integrated moisture recycling vulnerability. In the discussion, we further explore the megacity-specific nuance of how this vulnerability is actually coupled to pre-existing water security.

## Results

### Megacity watersheds

**Fig 2:**
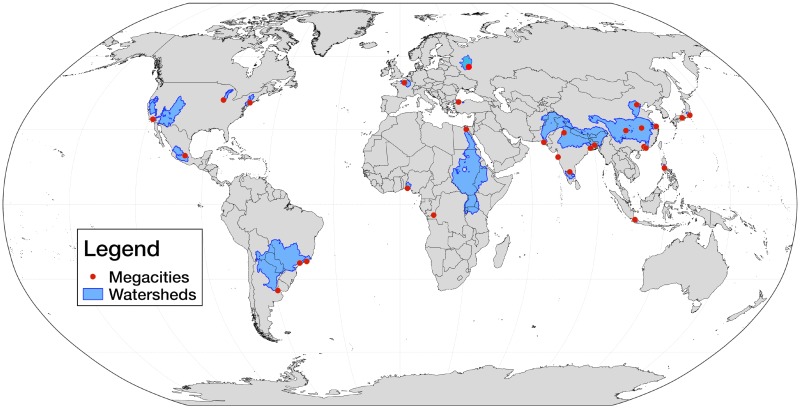
Global map of megacities, with corresponding watersheds that provide surface water and groundwater recharge. This figure is based on data from [3, 28], and was created by the authors using QGIS software.

There are 29 megacities globally, based on 2015 population data [3] (Fig 2). Of these, 26 cities were categorized as dependent on surface water for their water supplies (with water provided by direct rainfall runoff, snowmelt, or glacial melt), and the remaining three were primarily dependent on actively recharged groundwater. Several of the megacities obtained water from multiple watersheds, and we combined them into a single watershed. For the three groundwater-dependent megacities we identified the corresponding surface watersheds that contribute water to these groundwater capture zones, by overlaying the groundwater outflow points (from [3]) with the previously described database of global watersheds.

The current global distribution of megacities includes Africa, Eurasia, North and South America, located both along coasts and inland. There is also heterogeneity in the number of sources that comprise the different megacity watersheds, with some comprised of a single watershed (e.g. Cairo, Kinshasa), while others draw water from many different watersheds (e.g. Los Angeles, São Paulo). The watersheds depicted in Fig 2 became the sink regions used in our moisture tracking analysis.

### Precipitationshed identification

**Fig 3:**
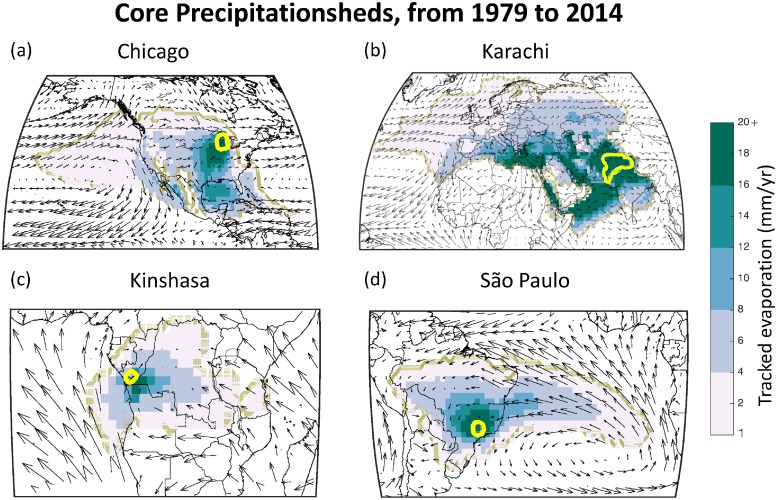
Megacity precipitationsheds, based on a core boundary (ranging from 1 mm/yr). Yellow lines enclose the sink regions, and the prevailing winds are indicated to illustrate the average direction of the winds throughout the year.

The precipitationsheds for all 29 megacities were calculated, and the moisture recycling details of these are summarized in Table 2. We depict a selection of the megacities in Fig 3, choosing cities that are (a) located on four different continents, (b) experience different rainy seasons, (c) have a range of watershed areas (with correspondingly, a range of precipitationshed areas), and (d) span the economic spectrum from low to high income. Note that in each figure the sink region (i.e. the megacity’s watershed) is indicated with a yellow line. The precipitationsheds for the remaining 25 megacities are included in the S1 Appendix.

**Table 2:** Summary of moisture recycling results for each of the 29 megacities, for neutral years only (i.e. not dry or wet years). The contribution columns indicate the amount of precipitation falling in the sink region (i.e. megacity watershed) that comes from that region, in terms of both the depth of precipitation falling in the sink region, and the fraction of annual precipitation that comes from that contributing region. Note, the ‘Watershed contribution’ column refers to internal moisture recycling within the sink region.

Megacity	Total Precip. (mm/yr)	Terrestiral Moisture Recycling ratio	Watershed contribution (aka Sink region)		Core Precipitationshed contribution (aka Source region)		Watershed + Core Precipitationshed contribution	
			depth (mm/yr)	fraction (%/yr)	depth (mm/yr)	fraction (%/yr)	depth (mm/yr)	fraction (%/yr)
Beijing, China	766	62%	49	6%	437	57%	486	63%
Bengaluru, India	833	25%	39	5%	62	7%	101	12%
Buenos Aires, Argentina	1,347	57%	309	23%	455	34%	764	57%
Cairo, Egypt	886	43%	183	21%	200	23%	382	43%
Chicago, USA	854	41%	26	3%	371	43%	397	47%
Chongqing, China	1,136	64%	163	14%	506	45%	669	59%
Delhi, India	732	43%	39	5%	147	20%	186	25%
Dhaka, Bangladesh	1,523	47%	279	18%	400	26%	679	45%
Guangzhou, China	1,741	36%	107	6%	442	25%	549	32%
Istanbul, Turkey	685	42%	26	4%	126	18%	153	22%
Jakarta, Indonesia	2,147	18%	52	2%	80	4%	132	6%
Karachi, Pakistan	676	49%	123	18%	222	33%	344	51%
Kinshasa, DRC	2,038	52%	75	4%	606	30%	681	33%
Kolkata, India	1,063	44%	161	15%	337	32%	498	47%
Lagos, Nigeria	1.479	51%	91	6%	471	32%	562	38%
Los Angeles, USA	406	29%	40	10%	33	8%	73	18%
Manila, Philippines	2,115	12%	42	2%	19	1%	62	3%
Mexico City, Mexico	694	32%	71	10%	97	14%	168	24%
Moscow, Russia	733	42%	20	3%	157	21%	176	24%
Mumbai, India	1,053	28%	15	1%	5	0%	20	2%
New York City, USA	1,178	37%	26	2%	296	25%	322	27%
Osaka-Kobe, Japan	1,510	29%	53	3%	128	8%	181	12%
Paris, France	844	28%	24	3%	57	7%	81	10%
Rio de Janeiro, Brazil	1,238	46%	46	4%	354	29%	400	32%
Sao Paulo, Brazil	1,304	54%	71	5%	566	43%	637	49%
Shanghai, China	1,203	37%	22	2%	207	17%	228	19%
Shenzhen, China	1,840	29%	38	2%	302	16%	340	18%
Tokyo, Japan	1,572	26%	23	1%	39	2%	62	4%
Wuhan, China	1,311	56%	176	13%	502	38%	678	52%

In a general sense, there are several features that are obvious from looking at the four panels in Fig 3. First, precipitationsheds can be very extensive in reach, in particular the precipitationsheds for Karachi and Chicago. Even for these two extensive precipitationsheds, however, the regions that contribute a lot of evaporation (relatively speaking) are much more concentrated near the sink region. Additionally, precipitationsheds can include regions that are a great distance from a sink region, while excluding areas that are very close to the sink region. For example, Chicago’s precipitationshed, includes contributions from the quite distant Pacific Ocean, while excluding regions that are nearby in Canada and the Northeastern USA.

The spatial patterns of the precipitationsheds are largely driven by the prevailing wind patterns. This is quite clear in the Chicago precipitationshed where we can see the flow of moisture from the southeastern United States and from the Gulf of Mexico. In São Paulo, we see the flow from the Atlantic, and how it piles against the Andes Mountains. We include the mean annual wind patterns in Fig 3 for reference.

Table 2 presents the full moisture recycling data for all of the megacities (rows), with columns for total annual precipitation, moisture recycling in the sink region (i.e. the megacity watershed), and the core precipitationshed (i.e. the land areas that contribute 1 mm/yr or more of evaporation to the precipitation in the sink region). There are several interesting details revealed in the summary table. First, there are a large number of cities that experience high terrestrial moisture recycling. Eight megacities receive around 50% or more of their watershed’s precipitation from upwind land areas, including Beijing, Buenos Aires, Chongqing, Karachi, Kinshasa, Lagos, São Paulo, and Wuhan. These could reasonably be considered “terrestrial moisture recycling-dependent” megacities, given their reliance on upwind land for sustaining their water supplies. There are also, four megacities that receive around 20% or more of their precipitation from internal moisture recycling, including Buenos Aires, Cairo, Dhaka, and Karachi. In other words, about 20% of the rain falling within each of these city’s watersheds originates as evaporation within that watershed. Finally, 8 of the 29 megacities receive nearly half of their precipitation from their core precipitationshed. Put differently, these 8 megacities are reliant on the land areas in their core precipitationshed (including the sink region itself) for providing evaporation to sustain their precipitation.

**Fig 4:**
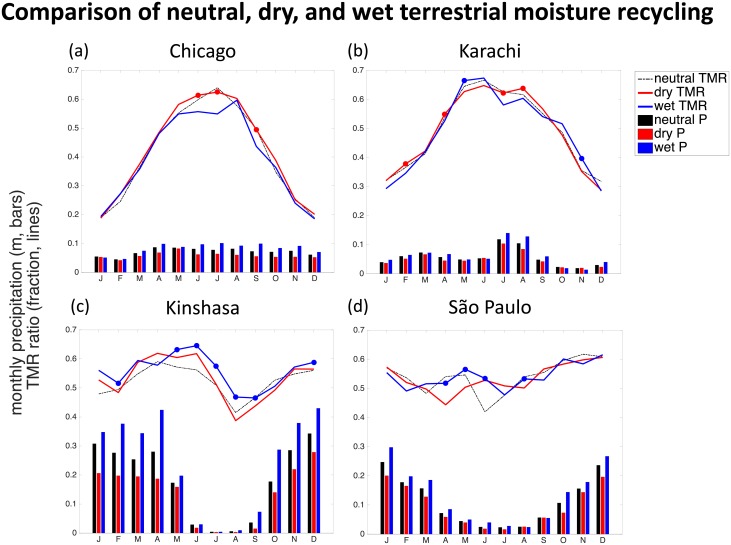
Summary of monthly average precipitation and terrestrial moisture recycling (TMR) during neutral, dry, and wet years. Note that the y-axis corresponds to both meters per month of precipitation (represented by bars), and the fraction of precipitation originating from upwind land surfaces (represented by lines). The dots indicate significant differences for either dry or wet years during that month; see Methods for further details.

The average characteristics presented above (Table 2) and the 36-year core precipitationshed (Fig 3), do not communicate the seasonal variation within a year. Fig 4 depicts the average monthly distribution of both precipitation in the megacity watersheds (bars) and the monthly average terrestrial moisture recycling (TMR) ratios (lines), for neutral, dry, and wet years.

In general, there is a wide range in the types of annual precipitation patterns we see among the four featured megacities (bars in Fig 4). There are three apparent types of annual cycles: relatively constant (e.g. Chicago), very wet season with very dry season (e.g. São Paulo, Kinshasa), and multiple rainy seasons (e.g. Karachi). The comparison of neutral, dry, and wet rainfall years (corresponding to black, red, and blue bars, respectively), indicates that in some locations there is very little difference between wet and dry years (e.g. Chicago, Karachi), whereas there are much bigger differences in others (e.g. São Paulo, Kinshasa).

The terrestrial moisture recycling ratios, referring to the fraction of rainfall coming from land versus ocean, are depicted as lines in Fig 4. An important note is that the three lines for neutral-, dry- and wet-year terrestrial moisture recycling do not follow the same patterns for each of the megacities. In some regions there is a peak in terrestrial moisture recycling (e.g. Chicago, Karachi), a relatively steady rate of moisture recycling (e.g. São Paulo), or increased variability in moisture recycling during the dry part of the year (e.g. Kinshasa).

The TMR ratios presented in Fig 4, suggest there may be significant differences between dry and wet year TMR. We found that 20 of 29 megacities had significantly higher TMR ratios during dry years, during two or more months, and ten of those experienced significant differences during four or more months. Conversely, eight megacities had significantly higher TMR during wet years for two or more months, and two of those had significantly higher TMR ratios during four months.

**Fig 5:**
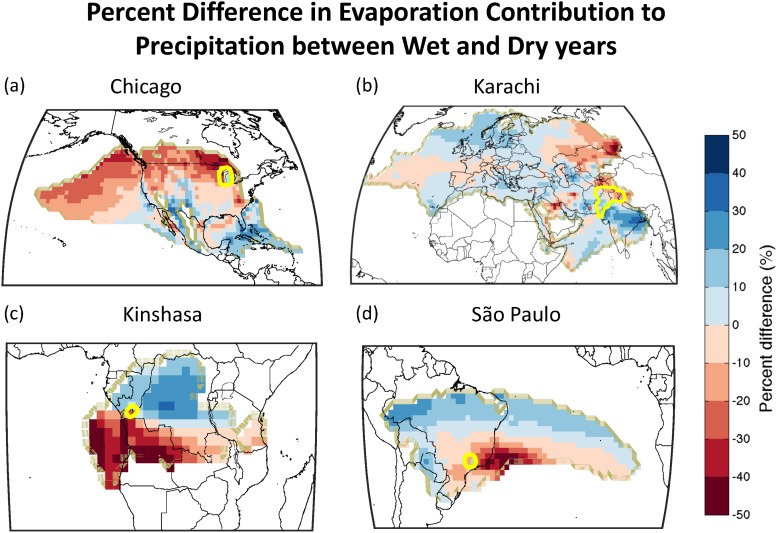
Percent difference between driest and wettest years of evaporation contribution to sink regions (yellow lines).

To explore the dry and wet year dynamics spatially, we calculated the difference in evaporation contribution during dry and wet years, and weighted each gridcell by its importance to megacity watershed rainfall (see Eq 3 in Methods). Fig 5 depicts this calculation for Chicago, Karachi, Kinshasa, and São Paulo. Chicago’s differences indicate more contribution from northern latitudes during dry years and more from lower latitudes in wet years. The relatively higher contributions from the Mexico-California region suggests wetter years may be associated with wet years in the desert southwest, and potentially with tropical storm activity in the Gulf of Mexico. Karachi’s differences are more heterogeneous than Chicago’s, but we still see the marked importance of land areas during dry years and oceanic sources during wet years. The Tibetan Plateau, Himalaya, and western Russia are key dry year sources of rainfall. Meanwhile, key wet year sources include the Indian subcontinent, parts of Iran, and southern Pakistan itself.

Kinshasa’s wet-year sources include much of the core of the Congo river basin. The dry-year sources originate in the Atlantic Ocean, as well as directly south from Angola and Zambia. São Paulo’s dry and wet year dynamics are quite complex, including ocean to land tele-connections. Dry year sources are dominated by relatively more contribution from just off the coast of São Paulo in the Atlantic Ocean, as well as just over São Paulo province itself. Wet year sources, however, are related to strong transport from the middle Atlantic Ocean, which then flows over the Amazon, piles against the Andes Mountains, and sweeps south to São Paulo. This tele-connection from the oceans to the Amazon is what makes wet years have significantly higher TMR ratios (Table 2), since moisture sources are predominantly in the southern Amazon, and central Brazil.

### Moisture recycling exposure

It is not a given that moisture recycling vulnerability will translate to urban water stress. There can be many buffers between precipitation falling in a watershed and the water that flows into a city’s pipes, including dam storage, transport via canals, and storage in local reservoirs. Our literature review of urban water supplies and exposure to moisture recycling dynamics revealed that many cities’ water supplies are in fact highly exposed to their moisture recycling. In some cases this was simply due to there being few buffers between moisture recycling dynamics and water supplies. We present the results of our moisture recycling exposure analysis below, with the results for the remaining 25 megacities provided in the S1 Appendix.

#### Chicago

Chicago receives its water from Lake Michigan, which is part of the network of the Great Lakes in the United States and Canada [34]. Lake Michigan is a precipitation and snowmelt dominated watershed, and withdrawals and management are very tightly regulated by both domestic regulations and international water law [34, 35]. The lake itself is highly polluted, and climate change may even threaten lake elevation leading to further challenges of water security in Chicago [35]. In Chicago the most relevant land-use change is conversion to agricultural land, especially in Canadian forests and prairies, which are disproportionately important during dry years. However, given that Lake Michigan, and the Great Lakes more generally, are massive, and given that there are several controls that can help manage the flow among the uppermost (Lake Superior) and lowest (Lake Ontario) lakes [36], we suggest that Chicago has a low exposure to moisture recycling dynamics.

#### Karachi

Karachi lies near the coast of Pakistan, and almost fully relies on water from the Indus River for its municipal supply [37]. However, the volume of water from the Indus is very insufficient for demand, and is made worse by a swelling population and inept management and institutions [38]. Karachi also has a very high reliance on a single source of surface runoff, a majority of which comes from either rain runoff or snowmelt [39]. Nearly 50% of the precipitation falling in the Indus comes from terrestrial sources with a pronounced reliance on the Tibetan plateau during dry years. Despite the natural buffer of glacial melt (which will likely diminish significantly in coming decades), Karachi has very few buffers for its water supply, suggesting that there is high exposure to moisture recycling dynamics.

#### São Paulo

São Paulo is reliant on surface runoff into a system of reservoirs called the Systema Cantareira [40, 41]. This network of reservoirs draws water from four different catchments in the São Paulo region. The storage in these reservoirs is quite sensitive to variations in rainfall [41], and thus there is limited buffer between changes in the delivery of rainfall and reservoir storage for supply. Additionally, pollution in the region is very high owing to high levels of informal and slum settlements adjacent to the reservoirs [40]. For São Paulo, the most relevant land-use change is likely to be in the zone of Brazil experiencing rapid conversion of tropical forest to grazing land and agricultural fields. These changes can lead to significant impacts for downwind precipitation, though there is debate over the magnitude of this impact (e.g. [20, 21]). There is much work being done on the topic of land-use change in Brazil, so water managers in São Paulo (as well as adjacent Rio de Janeiro) have a wealth of data and analysis to consider regarding water security challenges in the precipitationshed. Despite the concerted work to manage water issues (e.g. land-use change monitoring, water quality improvements, the management of limited supply), São Paulo’s reliance on rainfall that is primarily recycled from externally supplied sources suggests high exposure of São Paulo’s water supply and moisture recycling.

#### Kinshasa

Kinshasa’s water supply comes directly from the Ndjili River to the east of Kinshasa, which flows into the Congo River [42]. The river provides most of the municipal water supply to Kinshasa, and currently experiences high levels of contamination from human sewage [42–44]. Several treatment plants were installed historically, but have fallen into disrepair and cannot keep pace with population growth [43, 44]. Likewise, piped connections have broken, and overall municipal water service is unreliable and incomplete [44]. As for land-use change, Kinshasa is particularly vulnerable to changes in the interior of the Congo River basin, within the Democratic Republic of the Congo, and unlike some other megacities’ watersheds, the Congo is very understudied [42]. Given the lack of regulation of land-use, the direct use of water in the Ndjili river for drinking supply, and the inadequate infrastructure for managing changes in water, Kinshasa’s water supply is highly exposed to changes in moisture recycling [42–45].

### Moisture recycling vulnerability

Our results thus far include spatially mapping the sources of rainfall for each megacity’s watershed, and discovering the relatively higher importance of terrestrial sources of moisture during dry years. We take our analysis a step further to better understand how these realities about the precipitationshed interact with other aspects of water resources vulnerability, and find the answer to our third research question. The results reveal a wide range in vulnerability, with 4 highly vulnerable cities: Karachi, Shanghai, Wuhan, and Chongqing (Table 3). There are 6 medium and low vulnerability cities, and 4 very low vulnerability cities. All the megacities with a medium or high vulnerability score are currently experiencing water stress (*WS*). Also, perhaps not surprisingly, all the high-income countries (i.e. high economic capacity) have a low or very low vulnerability score. The moisture recycling indicators are less obvious in terms of the results, and we discuss this further in the following section. In terms of global distribution all of the highly and medium vulnerability megacities are located in Asia.

**Table 3:** Moisture recycling vulnerability analysis, as related to the megacity precipitationshed; ‘water stress’ (*WS*) and ‘economic capacity’ (*EC*) are taken from [3]; vegetation-regulated evaporation (*V*_*E*_) is taken from [23]; land-use change (*LUC*) is calculated using the HYDE 3.1 dataset (from [33]) and the precipitationsheds identified herein; ‘dry-year sensitivity’ (*DRY*) and ‘moisture recycling exposure’ (*MRE*) are calculated using the analysis herein. The order of the cities is from highest to lowest vulnerability.

MEGACITY	VULN.	*V*_*score*_	*WS*	*EC*	*MRE*	*V*_*E*_	*LUC*	*DRY*
Karachi, Pakistan	high	0.77	yes	low	100%	7.8%	-0.05%	yes
Shanghai, China	high	0.69	yes	medium	100%	7.6%	0.02%	yes
Wuhan, China	high	0.69	yes	medium	100%	5.0%	-0.01%	yes
Chongqing, China	high	0.62	yes	medium	100%	7.5%	-0.03%	yes
Delhi, India	medium	0.54	yes	low	100%	13.3%	0.08%	
Istanbul, Turkey	medium	0.54	yes	medium	100%	2.2%	-0.08%	yes
Shenzhen, China	medium	0.54	yes	medium	100%	2.9%	-0.02%	yes
Kolkata, India	medium	0.51	yes	low	33%	7.5%	-0.04%	yes
Beijing, China	medium	0.49	yes	medium	67%	7.8%	0.04%	yes
Moscow, Russia	medium	0.46	yes	medium	100%	4.0%	-0.19%	
Mexico City, Mexico	low	0.39	yes	medium	67%	0.0%	0.05%	yes
Kinshasa, DRC	low	0.38		very low	100%	10.5%	0.01%	
Mumbai, India	low	0.38		low	100%	0.0%	-0.34%	yes
New York City, USA	low	0.38		high	100%	6.1%	-0.07%	yes
Rio de Janeiro, Brazil	low	0.38	yes	medium	100%	3.5%	0.15%	
Dhaka, Bangladesh	low	0.33		very low	33%	4.9%	-0.03%	yes
Tokyo, Japan	low	0.31	yes	high	67%	0.0%	-0.06%	yes
Bengaluru, India	low	0.31	yes	low	100%	0.0%	-0.17%	
Lagos, Nigeria	low	0.31		low	100%	4.0%	0.13%	
Buenos Aires, Argentina	low	0.28		medium	67%	1.3%	0.06%	yes
Guangzhou, China	low	0.28		medium	67%	1.9%	0.06%	yes
Los Angeles, USA	low	0.23	yes	high	67%	0.0%	0.04%	
Jakarta, Indonesia	low	0.23		low	33%	0.0%	0.18%	yes
Manila, Philippines	low	0.23		low	33%	0.0%	0.57%	yes
Osaka-Kobe, Japan	low	0.21		high	67%	3.3%	-0.02%	yes
Cairo, Egypt	low	0.21		low	67%	3.7%	-0.02%	
Paris, France	very low	0.15		high	67%	0.0%	-0.04%	yes
Sao Paulo, Brazil	very low	0.15		medium	100%	4.9%	0.09%	
Chicago, USA	very low	0.10		high	33%	4.6%	0.05%	yes

### Vulnerability indicators

The *a priori* indicators of vulnerability (*water stress* and *economic capacity*) were weighted to reflect their importance in the actual vulnerability of megacities, but not so high as to overshadow the moisture recycling indicators. As such, the cities experiencing water stress are clustered in the top half of Table 3. However, economic capacity was not a key determinant of water supply vulnerability, though none of the most vulnerable cities have a high economic capacity.

There is very little pattern in the distribution of the moisture recycling indicators (*V*_*E*_, *LUC*, and *DRY*). This could be because moisture recycling exposure (*MRE*) is a very strong determinant of the eventual vulnerability score, and is not tied to the moisture recycling indicators directly, but rather the presence or absence of a megacity’s buffering capacity to mitigate moisture recycling change. Also, the *MRE* can only preserve or reduce the eventual vulnerability score, since it weights the moisture recycling indicators by a maximum of 1. The fact that there is less of an overall pattern in terms of moisture recycling indicators (in scanning from highest to lowest vulnerability) is not a weakness of the vulnerability score since it suggests that there is high heterogeneity in the types of moisture recycling dynamics experienced by megacities.

For example, Manila’s precipitationshed may be experiencing very rapid land-use change (*LUC*), but has almost no vegetation-regulated evaporation (*V*_*E*_), so that indicator is functionally zero. Similarly, Mumbai is sensitive to dry year moisture recycling, but *LUC* and *V*_*E*_ are sufficiently low to dampen the vulnerability score. Again, these interactions among the vulnerability indicators underscore the complexity of how changes in terrestrial moisture recycling may impact water security.

## Discussion

Cities that are reliant on surface water or actively recharged groundwater, are by extension reliant on the precipitation that falls in their watersheds. Referring back to our first research question, we find that 19 of 29 megacities receive more than a third of their water supplies from terrestrial moisture recycling, i.e. showing a dependence on land use upwind. Regarding our second research question, we find that for 20 of the 29 megacities, terrestrial moisture recycling is notably higher in dry years relative to wet years. Finally, for our third research question, we find that 4 of the 29 megacities experience high vulnerability related to their moisture recycling.

We unpack these results further below, in the context of geospatial perspectives and governance. We finish the discussion by examining pathways of future research and some of the limitations of our approach.

### Geospatial perspectives

The use of the megacity as the starting point for our analysis provided a focal point in terms of the number of people and the type of human footprint that is present on the land. However, there exists enormous variation in the spatial size of the corresponding watersheds, and subsequent precipitationsheds, for the 29 megacities. The reasons for this are that many urban settlements are ‘accidents’ of geography, hydrology, history, and climate (e.g. [46]), with some notable exceptions such as Shenzhen [47].

The range in size of precipitationsheds is vast, but the size of precipitationshed is not correlated with the size of the megacity. For example, one of the largest megacities on Earth (Tokyo) has one of the smallest precipitationsheds, simply because its watershed is also very small. This spatial variation is important since a large precipitationshed can itself provide a buffer to megacity water security, since even if the land-use change is a threat to downwind precipitation, the risk can be spread across a large area. Conversely, a small precipitationshed could be a particularly large risk, if land-use change is considerable (in either amount or speed of change). Likewise, if the areas of change are outside of the national boundaries or watershed boundaries, the ability to exert political or economic control on that land-use change may be limited.

The global distribution of the megacities is also important, with all of the high and medium vulnerability megacities located in Asia. This is not necessarily consistent with existing work on global distribution of water insecurity, since Africa is often considered the most water insecure continent (e.g. [48]). The importance of management and governance of land and water resources will be explored further below.

### Moisture recycling and governance

Several of the larger precipitationsheds in this study experience significant moisture recycling within the sink region itself. Cairo, Karachi, Dhaka, and Buenos Aires all receive nearly 20% of their rainfall from within their watersheds (i.e. ‘internal moisture recycling’). Put another way, for these megacities in particular, land-use change within the watershed may be very important. For example, the Buenos Aires watershed encompasses the entire La Plata basin. Intense moisture recycling processes occur within the basin itself, not least because of its moist, tropical air. Though we do not discuss smaller-scale feedbacks of land-use change on moisture recycling, the scales of some of these megacity watersheds suggest that basin-scale efforts to coordinate and address land-use change impacts to surface water may be relevant to adjacent river basins, since land-use change in one watershed can significantly modify precipitation in other watersheds [4, 49].

Integrated Water Resources Management (IWRM) is an existing strategy that aims for holistic management of water resources across multiple stakeholders, within an entire watershed [50]. In the past, IWRM has been particularly oriented towards runoff, and how it is used among farmers, industry, and cities. Given the importance of internal moisture recycling in several megacity watersheds, IWRM may be a reasonable and appropriate entry point for landscape management, particularly oriented towards moisture recycling [51]. High internal moisture recycling, i.e. within a watershed, could be a source of resilience, but that depends on many factors including whether land-use change leads to changes in circulation patterns that could send evaporation out of the watershed (e.g. [17]). Future work could aim to better understand the scales of appropriate management and governance of moisture recycling as it relates to urban water vulnerability, and whether in some cases existing watersheds are good entry points for such management [52, 53]. Such work would need to be specific to a particular megacity (and its regional land and climate dynamics).

The geopolitics of internal moisture recycling management is also an important consideration, since the watersheds of many of these megacities cross political borders that are tense, if not volatile. For example, Karachi’s watershed stretches into the Himalaya, and includes areas managed by Nepal, China, Afghanistan, and India. The Nile basin, which is Cairo’s source of water, stretches as far south as the center of the African continent. However, the Blue Nile, the most important tributary to the Nile proper, originates in Ethiopia—a country with whom Egypt has tense relations regarding historic water management. As Ethiopia develops its own surface water resources, as well as continues dramatic land-use change, Egypt will need to plan accordingly regarding its surface and atmospheric water resources. Previous work began exploring the importance of transboundary moisture recycling governance [53], in terms of tailored strategies depending on terrestrial moisture recycling dynamics. However, much work remains to be done to better understand how megacities can play a role in managing these national and international issues.

### Future exploration of moisture recycling vulnerability

It is important to view our analysis as complimentary to other forms of water resources vulnerability analysis, and as highlighting aspects of the water cycle that are ignored in conventional vulnerability analyses. Future work that aims to explore moisture recycling vulnerability could refine our vulnerability analysis in several ways, including different representations of vegetation-regulated moisture recycling and land-use change. The concept of vegetation-regulated moisture recycling is very recent [23], and thus there is a great deal of work to be done to improve the estimation, simulation, and validation of this variable. Similarly, the importance of land-use change (i.e. how much can land-use change actually modify moisture recycling) is still a topic of much debate. Future work on the topic of integrated moisture recycling vulnerability could consider different values of vegetation-regulation, different land-use change simulations, and different moisture tracking or modeling schemes.

Additionally, climate change impacts to moisture recycling dynamics were not included here since we were looking at historic data only. However, climate change impacts may continue to be important for future moisture recycling vulnerability, especially as the climate starts to experience significant changes towards the middle of the 21st century. Changes to jet streams, storm tracks, El Niño events, and many other impacts of climate change will likely interact in important and significant ways with moisture recycling vulnerability [54]. For example, urban water demand will continue to increase over the coming decades as the climate changes. Thus, analyses that are oriented towards understanding how climate change might interact with future changes in megacity moisture recycling vulnerability, must consider integrated simulations of demographic, land-use, and climate change, since they will all interact with one another. Scenarios of *possible* change could be more useful than projections of *likely* change, since the complexity of the integrated factors will render any single simulation incorrect, but possible scenarios could provide valuable guidance forward [55].

### Limitations and uncertainties

The topic of moisture recycling has been largely absent from any discussion of urban water security, but this analysis will hopefully elevate the topic as worthy of consideration in the broader discussion of urban sustainability. Our results characterize existing and historic relationships between moisture recycling patterns, the role of vegetation in sustaining moisture recycling patterns, and recent trends in land-use change. We note we are focusing almost entirely on quantity issues, not quality, with regard to our vulnerability analysis. Also, it should be kept in mind that future land-use changes do not always follow current trends linearly. The results of the vulnerability analysis could change with the use of modified variables (e.g. different assumptions about land-use change), or new variables (e.g. climate change impacts to the storm tracks). Land-use change effects on evaporation can very depending on type of change, land management, vegetation, and climate [56–58]. Land-use change effects on precipitation can vary depending on scale [59], modulate rainfall triggering mechanisms through aerosol generation and boundary layer interactions, and can modify circulation patterns at the scale of monsoon systems [17]. Nevertheless, moisture recycling is often the dominating process at the regional scale and relevant as a first-order estimate of land-precipitation connections.

## Conclusion

We identified the precipitationsheds for 29 megacities globally. Of these megacity precipitationsheds, 19 of 29 megacities get more than a third of annual precipitation from terrestrial sources. We also found that 20 of the 29 megacities experience significantly more terrestrial moisture recycling during dry years as opposed to wet years. By combining our analysis with previous work, we find that the water supplies of 4 megacities are highly vulnerable, including Karachi, Shanghai, Wuhan, and Chongqing. Thus, our findings lead us to conclude that some megacities ought to consider the land-use change dynamics occurring within both their precipitationshed and watershed, so that they can better understand the interconnectedness of their own water security.

## Supporting information

S1 Appendix Moisture recycling results for 25 megacities. Appendix includes results for the 25 megacities, that are not included in the main text of the manuscript, including results for: (a) the core precipitationshed, (b) the analysis of moisture recycling exposure, (c) monthly precipitation and terrestrial moisture recycling ratios, and (d) comparison of dry and wet year precipitationsheds.
(PDF)

## References

[1] Grimm NB, Faeth SH., Golubiewski NE, Redman CL, Wu J, Bai X, et al Global change and the ecology of cities. Science. 2008 doi: 10.1126/science.1150195 10.1126/science.1150195 18258902

[2] Jenerette GD, Larsen L. A global perspective on changing sustainable urban water supplies. Glob Planet Change. 2006.

[3] McDonald RI, Weber K, Padowski J, Flörke M, Schneider Ct, Green P A, et al Water on an urban planet: Urbanization and the reach of urban water infrastructure. Glob Environ Change. 2014 doi: 10.1016/j.gloenvcha.2014.04.022

[4] Wang-Erlandsson L, Fetzer I, Keys PW, van der Ent RJ, Savenije HH, Gordon LJ. Remote land use impacts on river flows through atmospheric teleconnections. Hydrol Earth Syst Sc Discussions. 2017 doi: 10.5194/hess-2017-494

[5] Gleeson T, Smith L, Moosdorf N, Hartmann J, Dürr HH, Manning AH et al Mapping permeability over the surface of the Earth. Geophys Res Lett,. 2011 doi: 10.1029/2010GL045565

[6] Lattemann S, Höpner T. Environmental impact and impact assessment of seawater desalination. Desalination. 2008 doi: 10.1016/j.desal.2007.03.009

[7] Linsley RK Jr, Kohler MA, Paulhus JLH. Hydrology for Engineers McGraw–Hill Series in Water Resources and Environmental Engineering. McGraw-Hill, Inc. 1982

[8] Taylor RG. Ground water and climate change. Nat Clim Change. 2012.

[9] Dirmeyer PA, Brubaker KL. Contrasting evaporative moisture sources during the drought of 1988 and the flood of 1993. J Geophys Res-Atmos. 1999 104(D16), 19383–19397 doi: 10.1029/1999JD900222

[10] Martinez JA, Dominguez F. Sources of Atmospheric Moisture for the La Plata River Basin. J Climate. 2014 doi: 10.1175/JCLI-D-14-00022.1

[11] van der Ent RJ, Savenije HHG, Schaefli B, Steele-Dunne SC. Origin and fate of atmospheric moisture over continents. Water Resour Res. 2010 doi: 10.1029/2010WR009127

[12] Keys PW, van der Ent RJ, Gordon LJ, Hoff H, Nikoli R, Savenije HHG. Analyzing precipitationsheds to understand the vulnerability of rainfall dependent regions. Biogeosciences. 2012 doi: 10.5194/bg-9-733-2012

[13] Keys PW, Barnes EA., van der Ent RJ, Gordon LJ. Variability of moisture recycling using a precipitationshed framework. Hydrol Earth Syst Sc. 2014 doi: 10.5194/hessd-11-5143-2014

[14] Bagley J. E., Desai A. R., Dirmeyer P. A, Foley J. A. Effects of land cover change on moisture availability and potential crop yield in the world’s breadbaskets. Environ Res Lett. 2012 doi: 10.1088/1748-9326/7/1/014009

[15] Salih AA, Körnich H, Tjernström M. Climate impact of deforestation over South Sudan in a regional climate model. Int J Climatol. 2013 doi: 10.1002/joc.3586

[16] Lo MH, Famiglietti JS. Irrigation in California’s Central Valley strengthens the southwestern US water cycle. Geophys Res Lett. 2013 doi: 10.1002/grl.50108

[17] Tuinenburg OA, Hutjes RWA, Stacke T, Wiltshire A, Lucas-Picher P. Effects of irrigation in India on the atmospheric water budget. J Hydrometeorol. 2014 doi: 10.1175/JHM-D-13-078.1

[18] Wei J, Dirmeyer PA, Wisser D, Bosilovich MG, Mocko DM. Where Does the Irrigation Water Go? An Estimate of the Contribution of Irrigation to Precipitation Using MERRA. J Hydrometeorol. 2013 doi: 10.1175/JHM-D-12-079.1

[19] Spracklen D. V., Garcia-Carreras L. The impact of Amazonian deforestation on Amazon basin rainfall. Geophys Res Lett. 2015 doi: 10.1002/2015GL066063

[20] Swann AL, Longo M, Knox RG, Lee E, Moorcroft PR. Future deforestation in the Amazon and consequences for South American climate. Agr Forest Meteorol. 2015 doi: 10.1016/j.agrformet.2015.07.006

[21] Badger AM, Dirmeyer PA. Climate response to Amazon forest replacement by heterogeneous crop cover. Hydrol Earth Syst Sc. 2015 doi: 10.5194/hess-19-4547-2015

[22] Halder S, Saha SK, Dirmeyer PA, Chase TN, Goswami BN. Investigating the impact of land-use land-cover change on Indian summer monsoon daily rainfall and temperature during 1951-2005 using a regional climate model. Hydrol Earth Syst Sc. 2016 doi: 10.5194/hess-20-1765-2016

[23] Keys PW, Wang-Erlandsson L, Gordon L.J. Revealing Invisible Water: Moisture Recycling as an Ecosystem Service. PloS one. 2016 doi: 10.1371/journal.pone.0151993 10.1371/journal.pone.0151993 PMC4801336 26998832

[24] Ellison D, Futter MN, Bishop K. On the forest cover-water yield debate: from demand- to supply-side thinking. Glob Change Biol. 2012 doi: 10.1111/j.1365-2486.2011.02589.x

[25] Bagley JE, Desai AR, Harding KJ, Snyder PK, Foley JA. Drought and deforestation: has land cover change influenced recent precipitation extremes in the Amazon?. J Climate. 2014 doi: 10.1175/JCLI-D-12-00369.1

[26] Fernandes K, Giannini A, Verchot L, Baethgen W, Pinedo-Vasquez M. Decadal covariability of Atlantic SSTs and western Amazon dry-season hydroclimate in observations and CMIP5 simulations. Geophys Res Lett. 2015 doi: 10.1002/2015GL063911

[27] Spracklen DV, Arnold SR, Taylor CM. Observations of increased tropical rainfall preceded by air passage over forests. Nature. 2012 doi: 10.1038/nature11390 22951966 10.1038/nature11390

[28] ESRI. ArcGIS Desktop: Release 9.3. Redlands, CA: Environmental Systems Research Institute. 2009

[29] Stohl A, Hittenberger M, Wotawa G. Validation of the lagrangian particle dispersion model FLEXPART against large-scale tracer experiment data. Atmos Environ. 1998 doi: 10.1016/S1352-2310(98)00184-8

[30] Dominguez F, Kumar P, Liang XZ, Ting M. Impact of atmospheric moisture storage on precipitation recycling. J Climate. 2006 doi: 10.1175/JCLI3691.1

[31] van der Ent RJ, Wang-Erlandsson L, Keys PW, Savenije HHG. Contrasting roles of interception and transpiration in the hydrological cycle—Part 2: Moisture recycling. Earth Syste Dynam. 2014 doi: 10.5194/esdd-5-281-2014

[32] Dee DP, Uppala SM, Simmons AJ, Berrisford P, Poli P, Kobayashi S et al The ERA-Interim reanalysis: Configuration and performance of the data assimilation system. Q J Roy Meteor Soc. 2011 doi: 10.1002/qj.828

[33] Klein Goldewijk K, Beusen A, Van Drecht G, De Vos M. The HYDE 3.1 spatially explicit database of human-induced global land-use change over the past 12,000 years. Global Ecol Biogeogr. 2011 doi: 10.1111/j.1466-8238.2010.00587.x

[34] Hayhoe K, VanDorn J, Croley T II, Schlegal N, Wuebbles D. Regional climate change projections for Chicago and the US Great Lakes. J Great Lakes Res. 2010.

[35] Allan JD, McIntyre PB, Smith SD, Halpern BS, Boyer GL, Buchsbaum A, et al Joint analysis of stressors and ecosystem services to enhance restoration effectiveness. Proc Natl Acad Sci USAof Sciences. 2013 doi: 10.1073/pnas.1213841110 10.1073/pnas.1213841110 PMC3538252 23248308

[36] Burnett AW, Kirby ME, Mullins HT, Patterson WP. Increasing Great Lake-effect snowfall during the twentieth century: A regional response to global warming?. J Climate. 2003 16(21), 3535–3542. doi: 10.1175/1520-0442(2003)016%3C3535:IGLSDT%3E2.0.CO;2

[37] Rahman A, Lee HK, Khan MA. Domestic water contamination in rapidly growing megacities of Asia: Case of Karachi, Pakistan. Environ Monit Assess. 1997 44(1-3), 339–360. doi: 10.1023/A:1005747732104

[38] Mustafa D, Akhter M, Nasrallah N. Understanding Pakistan’s water-security nexus. Washington, DC: United States Institute of Peace 2013.

[39] Lutz AF, Immerzeel WW, Kraaijenbrink PDA, Shrestha AB, Bierkens MF. Climate change impacts on the upper indus hydrology: sources, shifts and extremes. PloS one. 2016 doi: 10.1371/journal.pone.0165630 10.1371/journal.pone.0165630 PMC5102348 27828994

[40] Formiga Johnsson, RM, Kemper KE. Institutional and Policy Analysis of River Basin Management: The Alto Tiete River Basin, São Paulo, Brazil. World Bank Policy Research Working Paper No. 3650. 2005. Available from: http://documents.worldbank.org/curated/en/403111468229455654/pdf/wps3650.pdf

[41] Coutinho RM, Kraenkel RA, Prado PI. Catastrophic regime shift in water reservoirs and São Paulo water supply crisis. PloS one. 2015 doi: 10.1371/journal.pone.0138278 10.1371/journal.pone.0138278 PMC4570716 26372224

[42] Ndolo Goy, P. GIS-based soil erosion modeling and sediment yield of the Ndjili River basin, Democratic Republic of Congo. Masters dissertation, Colorado State University. 2015.

[43] USAID. Democratic Republic of the Congo: Water and Sanitation Profile. USAID. 2008. Available from: http://pdf.usaid.gov/pdf_docs/Pnado929.pdf

[44] United Nations Environment Programme. Water Issues in the Democratic Republic of the Congo. UNEP. 2011. Available from: http://postconflict.unep.ch/publications/UNEP_DRC_water.pdf

[45] Food and Agriculture Organization. Country Fact Sheet: Democratic Republic of Congo. FAO. 2017. Available from: http://www.fao.org/nr/water/aquastat/data/cf/readPdf.html?f=COD-CF_eng.pdf

[46] Black D, Henderson V. Urban evolution in the USA. J Econ Geogr. 2003 3(4), 343–372. doi: 10.1093/jeg/lbg017

[47] Güneralp B, Seto KC. Environmental impacts of urban growth from an integrated dynamic perspective: A case study of Shenzhen South China. Glob Env Change. 2008.

[48] Gain AK, Giupponi C, Wada Y. Measuring global water security towards sustainable development goals. Environ Res Lett. (2017)

[49] Weng W, Luedeke MKB, Zemp DC, Lakes T, Kropp JP. Aerial and surface rivers: downwind impacts on water availability from land use changes in Amazonia. Hydrol Earth Syst Sci Discuss. In review.

[50] Al Radif A. Integrated water resources management (IWRM): An approach to face the challenges of the next century and to avert future crises. Desalination. 1999 doi: 10.1016/S0011-9164(99)00099-5

[51] Rockström J, Karlberg L, Wani SP, Barron J, Hatibu N, Oweis T, et al Managing water in rainfed agriculture-The need for a paradigm shift. Agr Water Manage.

[52] Giordano M, Shah T. From IWRM back to integrated water resources management. Int J Water Resour D. 2014 doi: 10.1080/07900627.2013.851521

[53] Keys PW, Wang-Erlandsson L, Gordon LJ, Galaz V, Ebbesson J. Approaching moisture recycling governance. Glob Environ Change. 2017 doi: 10.1016/j.gloenvcha.2017.04.007

[54] Gimeno L, Stohl A, Trigo RM, Dominguez F, Yoshimura K, Yu L, et al Oceanic and terrestrial sources of continental precipitation. Rev Geophys. 2012 doi: 10.1029/2012RG000389

[55] Merrie A, Keys P, Metian M, Österblom H. Radical Ocean Futures? scenario development using science fiction prototyping. Futures. 2017.

[56] Negri AJ, Adler RF, Xu L, Surratt J. The impact of Amazonian deforestation on dry season rainfall. J Climate 17, no. 6 (2004), 1306–1319. doi: 10.1175/1520-0442(2004)017%3C1306:TIOADO%3E2.0.CO;2

[57] Teuling AJ, Seneviratne SI, Stöckli R, Reichstein M, Moors E, Ciais P, et al Contrasting response of European forest and grassland energy exchange to heatwaves. Nat Geosci. 2010 doi: 10.1038/ngeo950

[58] Lawrence D, Vandecar K. Effects of tropical deforestation on climate and agriculture. Nat Clim Change. 2015 doi: 10.1038/nclimate2430

[59] D’Almeida C, Vörösmarty CJ, Hurtt GC, Marengo JA, Dingman SL, Keim BD. The effects of deforestation on the hydrological cycle in Amazonia: a review on scale and resolution. Int J Climatol. 2007.

