# Supplementary material for: Megacity precipitationsheds reveal tele-connected water security challenges
Source: PLoS One. 2018 Mar 13;13(3):e0194311. doi: 10.1371/journal.pone.0194311 (PMC5849328; doi:10.1371/journal.pone.0194311)
Supplement: S1 Appendix — Appendix includes results for the 25 megacities, that are not included in the main text of the manuscript, including results for: (a) the core precipitationshed, (b) the analysis of moisture recycling exposure, (c) monthly precipitation and terrestrial moisture recycling ratios, and (d) comparison of dry and wet year precipitationsheds. (PDF) [file pone.0194311.s001.pdf]

S1 Appendix for *Megacity precipitationsheds reveal tele-connected water security challenges*

1. Moisture recycling results for 25 megacities

The results of our analysis for the megacities not featured in the main manuscript are presented here. For each of the megacities there are four components:

a) Core precipitationshed (as seen in Fig 3 in manuscript)

b) Analysis of moisture recycling exposure (used in vulnerability analysis), with references

c) Monthly precipitation & terrestrial moisture recycling ratios (as seen in Fig 4 in manuscript)

d) Comparison between dry and wet year precipitationsheds (as seen in Fig 5 in manuscript)

Beijing, China

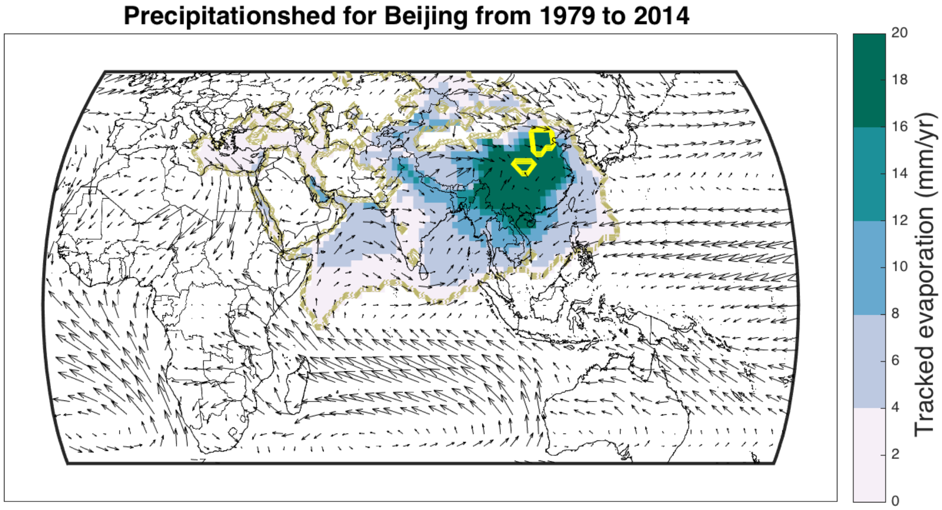

Figure S1: Beijing precipitationshed, based on a core boundary (ranging from 1 mm/yr). Yellow lines enclose the sink region, and the prevailing winds are indicated to illustrate the average direction of the winds throughout the year.

Moisture recycling exposure analysis

Beijing is one of the most water scarce countries in the world. Limited surface water, and decreasing trends in precipitation and corresponding runoff has led to unsustainable groundwater extraction being used to meet around two thirds of municipal water demand. Surface reservoirs operate at well below design capacity due to decreasing inflow, while pollution has rendered some historically-used reservoirs unsafe. To complement local supply, additional water is transported via the South North Water Transfer project, from the Han River, to the south. The fact that the groundwater aquifer ultimately relies on recharge, that the surface reservoirs are operating below capacity, means that there is little margin for error in water management yet some buffer between runoff and aquifer recharge. We suggest that Beijing's water security is moderately exposed to moisture recycling dynamics.

References

1. Xia, J, Zhang, L, Liu, C, Yu, J. Towards better water security in North China. Water Resources Management. 2007. doi: 10.1007/s11269-006-9051-1

2. Zheng, C, Liu, J, Cao, G, Kendy, E, Wang, H, Jia, Y. Can China cope with its water crisis? - Perspectives from the North China Plain. Groundwater. 2010. 48(3), 350-354.

3. Beijing's Water Crisis 1949 - 2008 Olympics. Probe International Beijing Group. June 2008.

Beijing, China (*continued*)

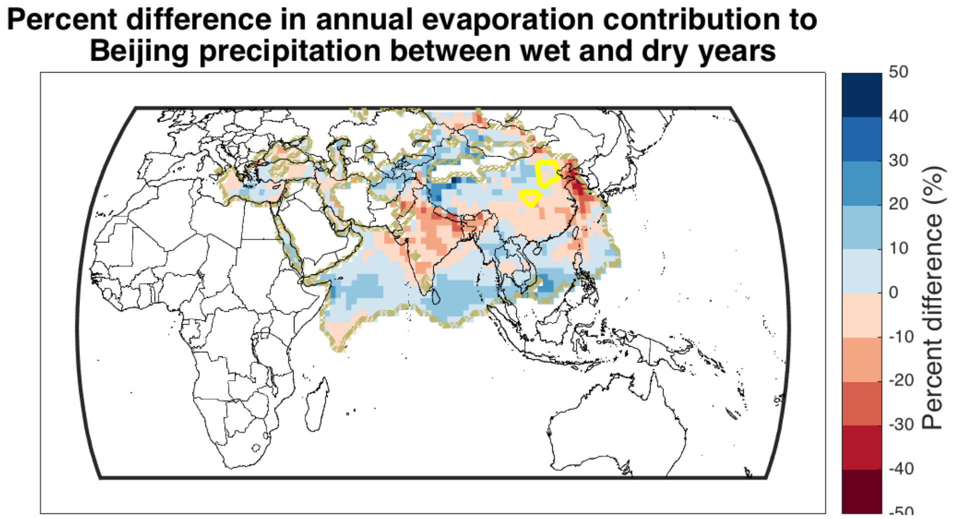

Figure S2: Percent difference in evaporation contribution between driest and wettest years.

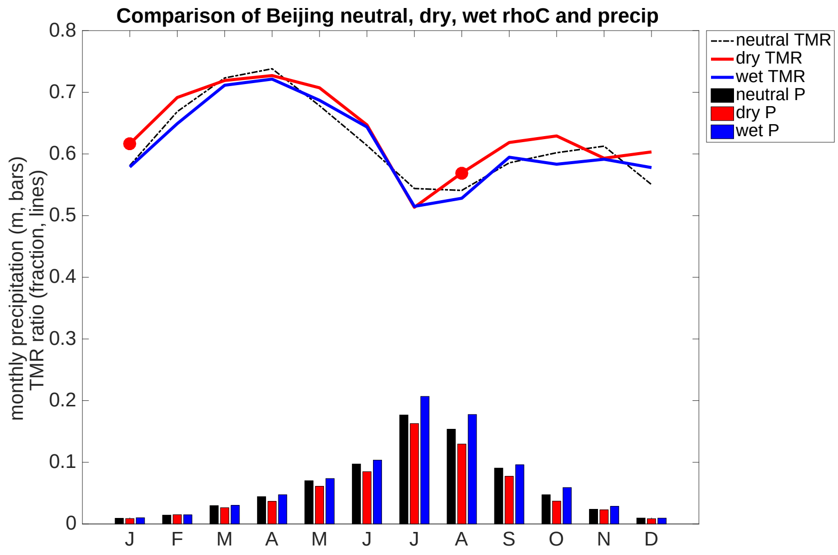

Figure S3: Summary of monthly average precipitation and terrestrial moisture recycling (TMR) during neutral, dry, and wet years. Note that the y-axis corresponds to both m/month of precipitation (represented by bars), and the fraction of precipitation originating from upwind land surfaces (represented by lines). The dots indicate significant differences for either dry or wet years during that month; see Methods in main article for further details.

Bengaluru, India

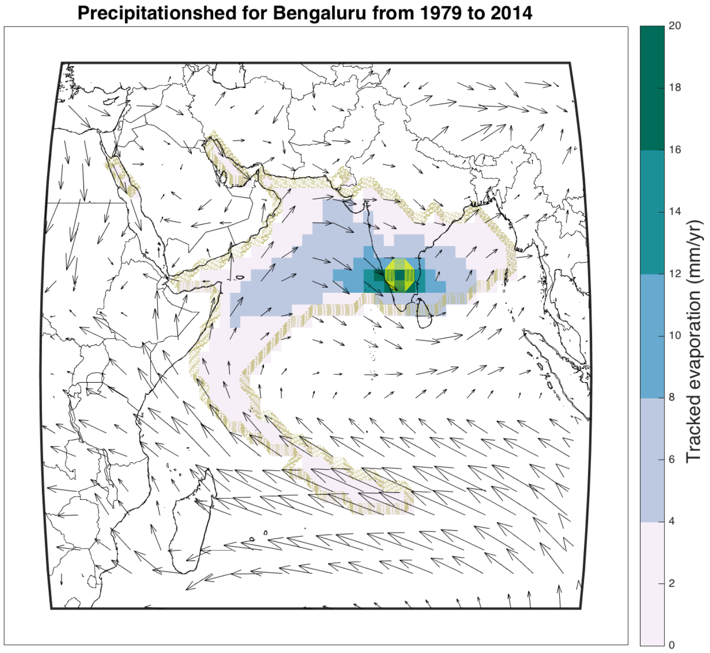

Figure S4: Bengaluru precipitationshed, based on a core boundary (ranging from 1 mm/yr). Yellow lines enclose the sink region, and the prevailing winds are indicated to illustrate the average direction of the winds throughout the year.

Moisture recycling exposure analysis

Bengalaru is surface water dependent, receiving 80% of water from the Cauvery river, and 20% from the Arkavathy river. Both sources of supply are erratic in their supply, with the Arkavathy suffering decreasing rainfall availability. A complex, engery-intensive network of pipes and pumps brings water uphill to Bengalaru from these reservoirs, but a growing population makes sufficient infrastructure investment difficult. Given the utter reliance on relatively small runoff-dominated basins, with limited buffer in either groundwater, inter-annual reservoir storage, or snowmelt, this megacity is highly exposed to its moisture recycling dynamics.

References

1. Das, S. Water security in Bengaluru. Journal of the Geological Society of India. 2013. 81(5), 595-599.

2. Srinivasan, V, Thompson, S, Madhyastha, K, Penny, G, Jeremiah, K, Lele, S. Why is the Arkavathy River drying? A multiple-hypothesis approach in a data-scarce region. Hydrology and Earth System Sciences. 2015. doi:10.5194/hess-19-1905-2015

3. Enqvist, J, Tengö, M, Boonstra, WJ. Against the current: rewiring rigidity trap dynamics in urban water governance through civic engagement. Sustainability Science. 2016. doi: 10.1007/s11625-016-0377-1

Bengaluru, India (*continued*)

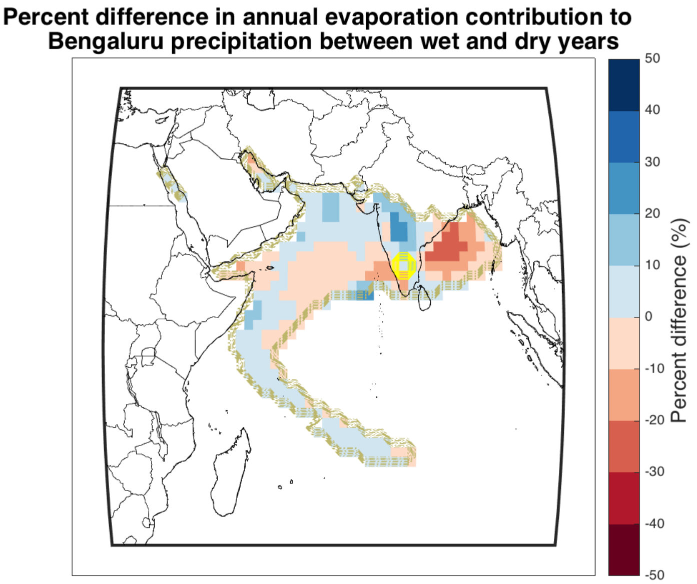

Figure S5: Percent difference in evaporation contribution between driest and wettest years.

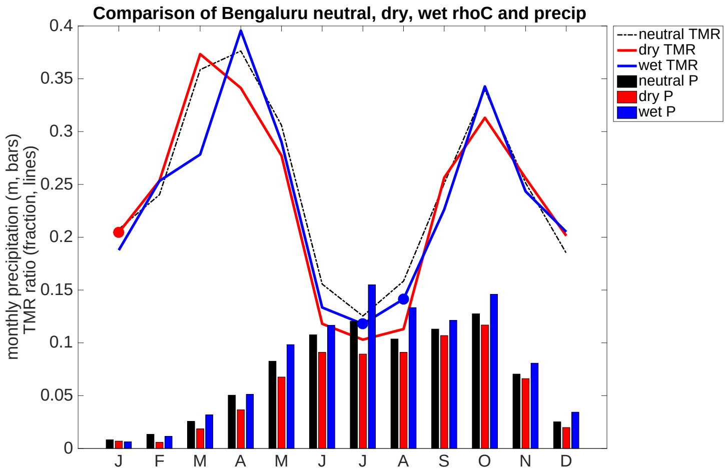

Figure S6: Summary of monthly average precipitation and terrestrial moisture recycling (TMR) during neutral, dry, and wet years. Note that the y-axis corresponds to both m/month of precipitation (represented by bars), and the fraction of precipitation originating from upwind land surfaces (represented by lines). The dots indicate significant differences for either dry or wet years during that month; see Methods in main article for further details.

Buenos Aires, Argentina

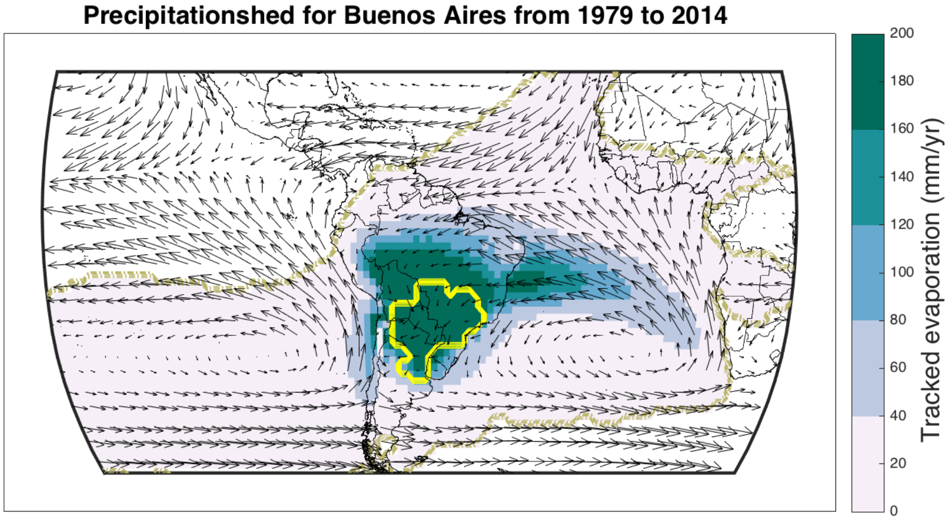

Figure S7: Buenos Aires precipitationshed, based on a core boundary (ranging from 1 mm/yr). Yellow lines enclose the sink region, and the prevailing winds are indicated to illustrate the average direction of the winds throughout the year.

Moisture recycling exposure analysis

Buenos Aires exists at the mouth of the Rio de La Plata, which provides around 95% of the municipal water demand for Buenos Aires. Due to lack of sewage treatment, but especially heavy industrial contamination, the La Plata is the most polluted water body in Argentina. While moisture recycling is high, including average internal moisture recycling of 23%, reliance on treated surface water for municipal supply provides a buffer against changes in moisture recycling dynamics. Thus, we suggest this megacity is moderately exposed to its moisture recycling dynamics.

References

1. Engel, K, Jokiel, D, Kraljevic, A, Geiger, M, Smith, K. "Big Cities, Big Water, Big Challenges: Water in an Urbanizing World". 2011. World Wildlife Fund: 25-32.

2. Kane, S. Water Security in Buenos Aires and the Paran-Paraguay Waterway. Human Organization. 2012. doi: 10.17730/humo.71.2.b884qx914u101j42

3. Loftus, AJ, McDonald, DA . Of liquid dreams: a political ecology of water privatization in Buenos Aires. Environment and Urbanization. 2001. 13(2), 179-199.

Buenos Aires, Argentina (*continued*)

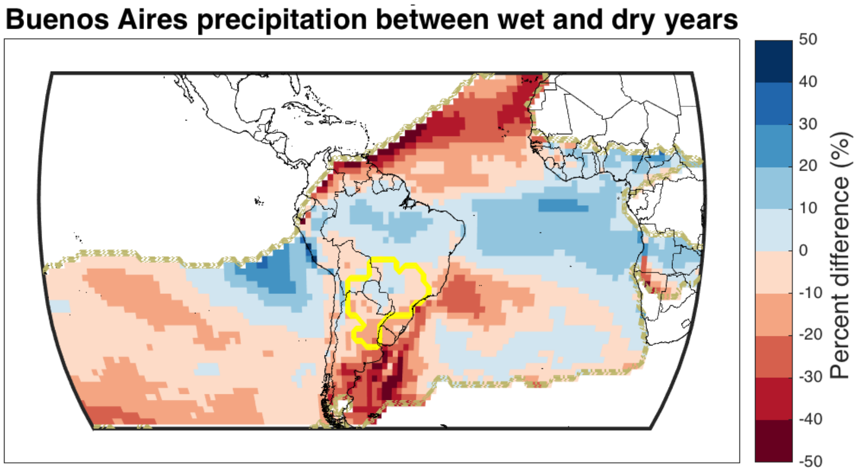

Figure S8: Percent difference in evaporation contribution between driest and wettest years.

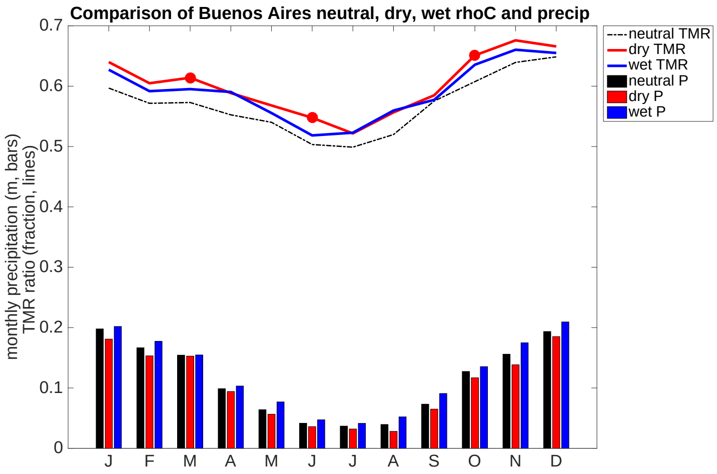

Figure S9: Summary of monthly average precipitation and terrestrial moisture recycling (TMR) during neutral, dry, and wet years. Note that the y-axis corresponds to both m/month of precipitation (represented by bars), and the fraction of precipitation originating from upwind land surfaces (represented by lines). The dots indicate significant differences for either dry or wet years during that month; see Methods in main article for further details.

Cairo, Egypt

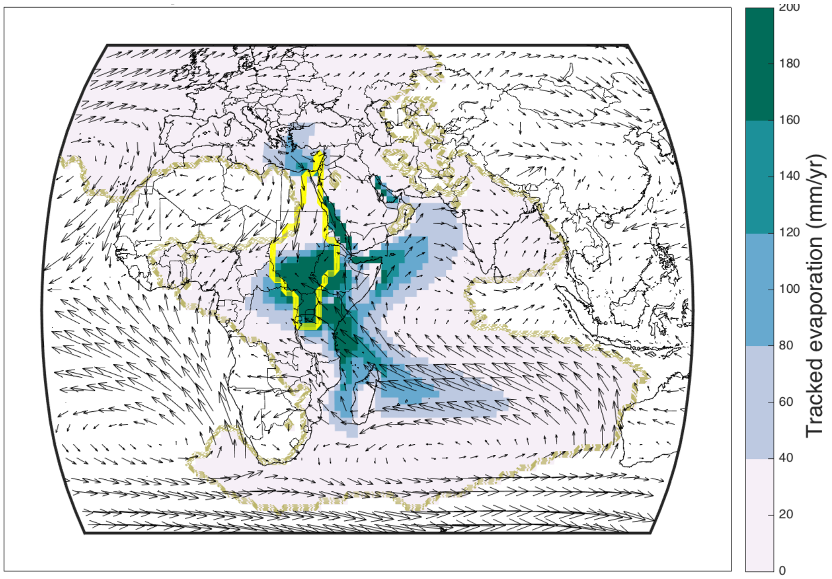

Figure S10: Cairo precipitationshed, based on a core boundary (ranging from 1 mm/yr). Yellow lines enclose the sink region, and the prevailing winds are indicated to illustrate the average direction of the winds throughout the year.

Moisture recycling exposure analysis

Cairo exists near the mouth of the Nile River, from which it receives nearly all of its water supply. The Aswan High Dam in southern Egypt stores approximately 1.5 times the flow of the Nile River, providing a very large water supply buffer. Despite a desert climate, and the potential decrease in Nile River flow from upstream riparians, municipal water use represents a small fraction of total water use in Egypt (around 8%). Thus, the water security of Cairo has some buffer, i.c. shifting water uses and adequate storage. However, the uncertainty of upstream Nile Basin country behavior (including Ethiopia which is constructing a very large dam that will retain a significant fraction of the Blue Nile), and the reliance on the Nile for surface runoff generated by significant internal moisture recycling within the Nile watershed itself, suggests that Cairo is moderately exposed to its moisture recycling dynamics.

References

1. Martin Hvidt: Water resource planning in Egypt, in: Eric Watkins (Editor):The Middle Eastern Environment, ISBN 1-898565-03-1, 1995

2. The Ministry of Water Resources and Irrigation; Arab Republic of Egypt (2005), Integrated Water Resources Management Plan.

3. El-Sadek, A. Water desalination: an imperative measure for water security in Egypt. Desalination. 2010. doi: 10.1016/j.desal.2009.09.143

Cairo, Egypt (*continued*)

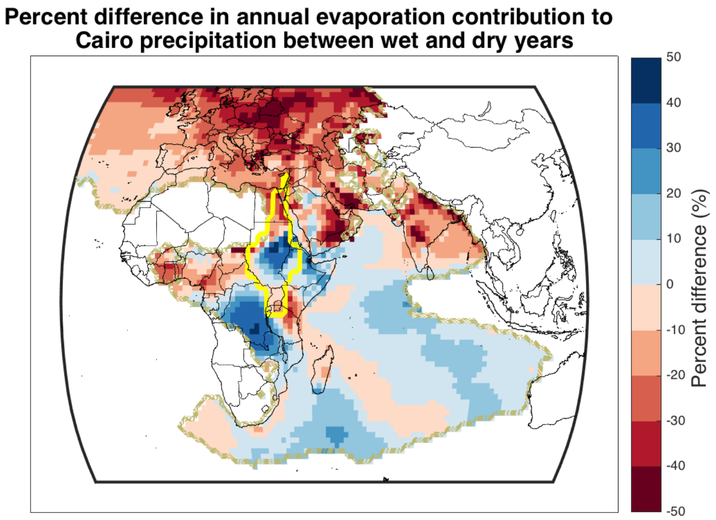

Figure S11: Percent difference in evaporation contribution between driest and wettest years.

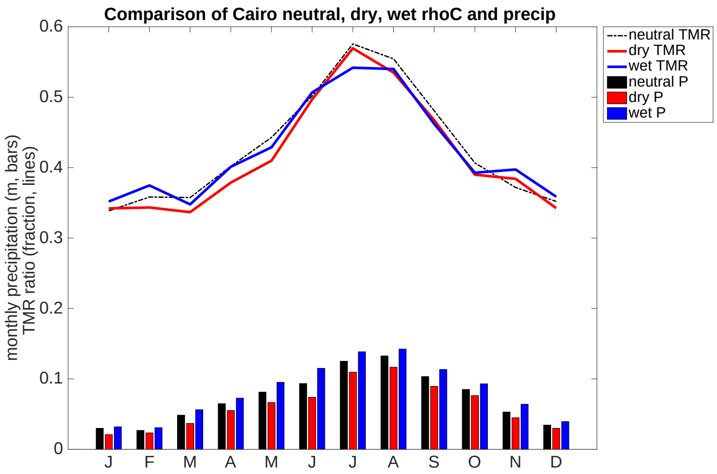

Figure S12: Summary of monthly average precipitation and terrestrial moisture recycling (TMR) during neutral, dry, and wet years. Note that the y-axis corresponds to both m/month of precipitation (represented by bars), and the fraction of precipitation originating from upwind land surfaces (represented by lines). The dots indicate significant differences for either dry or wet years during that month; see Methods in main article for further details.

Chongqing, China

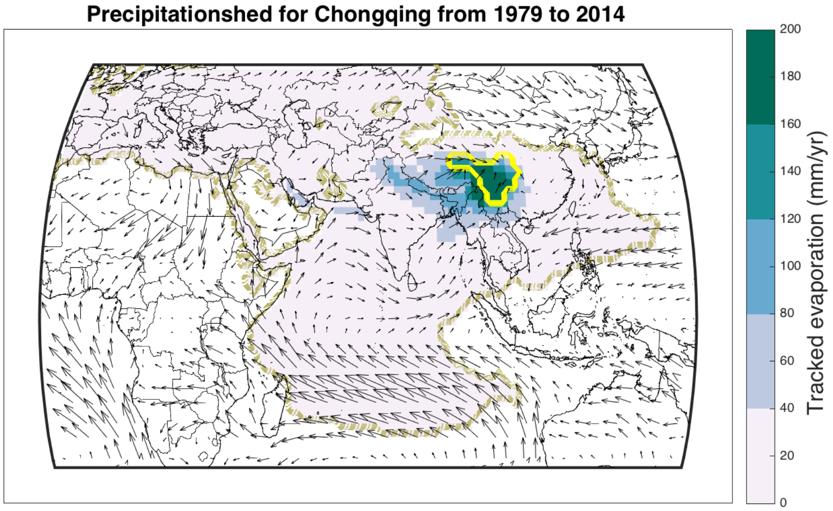

Figure S13: Chongqing precipitationshed, based on a core boundary (ranging from 1 mm/yr). Yellow lines enclose the sink region, and the prevailing winds are indicated to illustrate the average direction of the winds throughout the year.

Moisture recycling exposure analysis

Chongqinq is the primary city near the Three Gorges Dam on the Yangtze River. Despite the proximity to the dam, significant agricultural pollution and supply shortfalls plague the city. The primary issues of water security are rapid economic growth and demographic change, while a primary driver of stability in the water security is the forest cover in the basin. Given the dependence on surface water, the apparent importance of trees in helping regulate this water, and the importance of internal watershed moisture recycling, we consider Chongqing to be highly exposed to its moisture recycling dynamics.

References

1. Zhang, J, Wang, L. Assessment of water resource security in Chongqing City of China: What has been done and what remains to be done? Natural Hazards. 2014. doi: 10.1007/s11069-014-1460-5

2. Ho, JY, Chan, EY, Guo, C, Liu, S, Yeung, MP. Association Between Water Security, Disaster Risk Perception and Preparedness Behavior of a Rural Ethnic Minority Village in Chongqing, China: A Pilot Study. Prehospital and Disaster Medicine. 2017. doi: 10.1017/S1049023X17003442

3. Zhang, C, Anadon, LD. A multi-regional input-output analysis of domestic virtual water trade and provincial water footprint in China. Ecological Economics. 2014. doi: 10.1016/j.ecolecon.2014.02.006

Chongqing, China (*continued*)

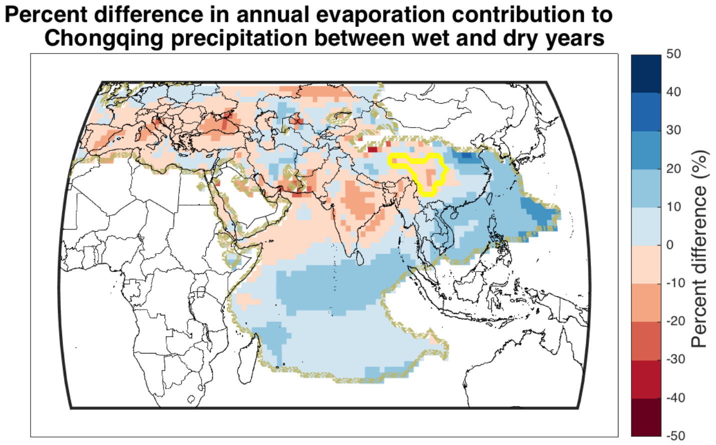

Figure S14: Percent difference in evaporation contribution between driest and wettest years.

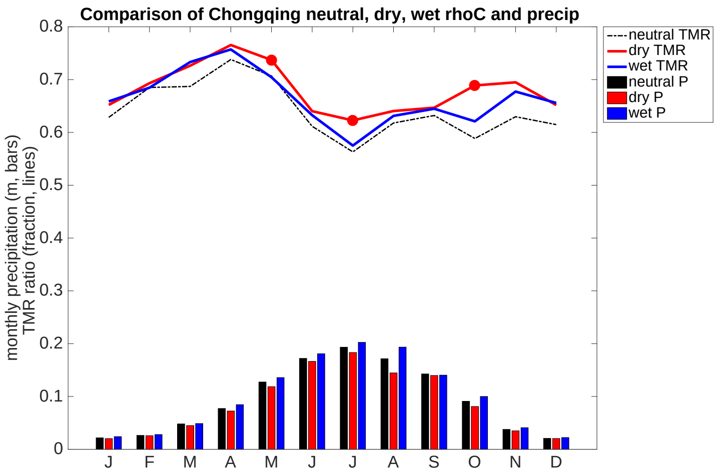

Figure S15: Summary of monthly average precipitation and terrestrial moisture recycling (TMR) during neutral, dry, and wet years. Note that the y-axis corresponds to both m/month of precipitation (represented by bars), and the fraction of precipitation originating from upwind land surfaces (represented by lines). The dots indicate significant differences for either dry or wet years during that month; see Methods in main article for further details.

Delhi, India

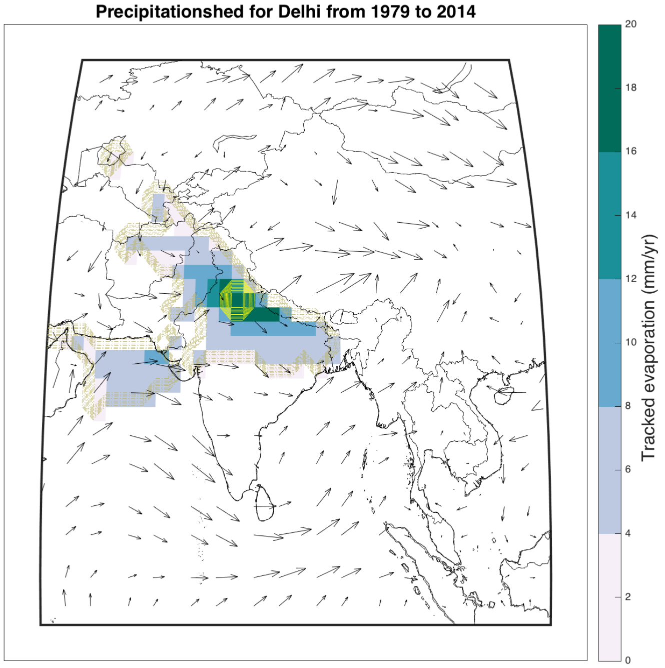

Figure S16: Delhi precipitationshed, based on a core boundary (ranging from 1 mm/yr). Yellow lines enclose the sink region, and the prevailing winds are indicated to illustrate the average direction of the winds throughout the year.

Moisture recycling exposure analysis

Delhi is a very water scarce city that relies primarily on runoff from the Yamuna River in Northern India. The Yamuna experiences relatively high moisture recycling, and the demand on the water from the Yamuna far exceeds the supply. In recent years there is strong evidence of very signifcant sensitivity of local water security to below-normal rainfall years, which has even led to security concerns of the canal that connects Delhi to important Yamuna reservoirs. Thus, the low storage buffer, the importance of terrestrial moisture recycling, and the high reliance on surface runoff, suggest that Delhi's water security is highly exposed to moisture recycling dynamics.

References

1. Soni, V, Gosain, AK, Datta, PS, Singh, D. A new scheme for large-scale natural water storage in the floodplains: the Delhi Yamuna floodplains as a case study. Current Science. 2009. 1338-1342.

2. Reuters. India's water reservoirs at 15 pct capacity in past week. U.S.A. 2017.

3. Rajawat, S.. "Drought And Water Security In India - Future Directions International". Future Directions International. 2016.

Delhi, India (*continued*)

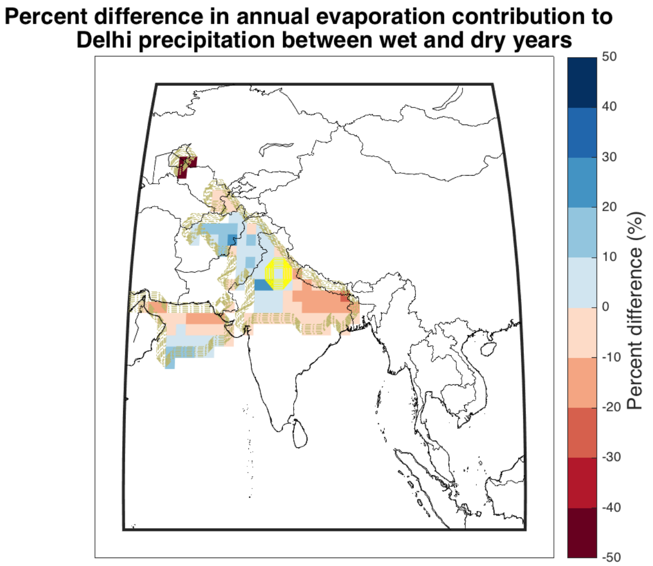

Figure S17: Percent difference in evaporation contribution between driest and wettest years.

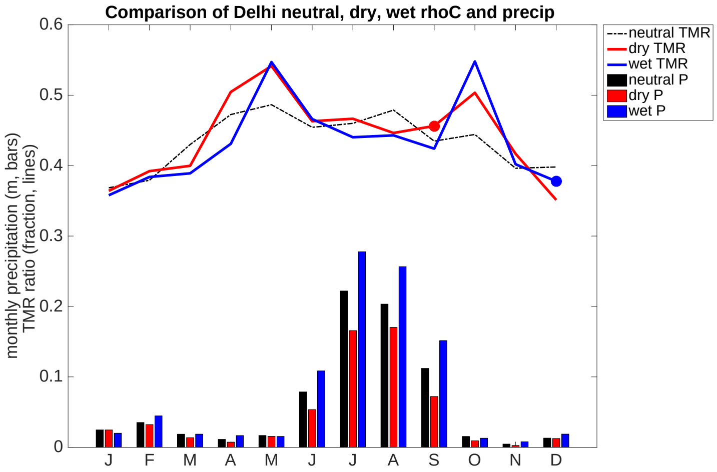

Figure S18: Summary of monthly average precipitation and terrestrial moisture recycling (TMR) during neutral, dry, and wet years. Note that the y-axis corresponds to both m/month of precipitation (represented by bars), and the fraction of precipitation originating from upwind land surfaces (represented by lines). The dots indicate significant differences for either dry or wet years during that month; see Methods in main article for further details.

Dhaka, Bangladesh

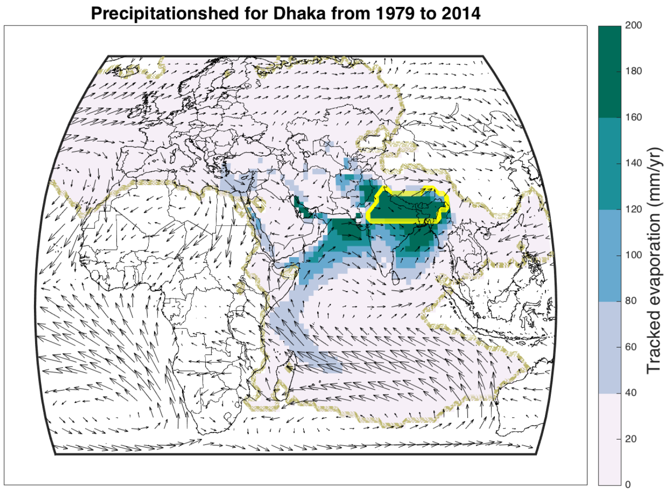

Figure S19: Dhaka precipitationshed, based on a core boundary (ranging from 1 mm/yr). Yellow lines enclose the sink region, and the prevailing winds are indicated to illustrate the average direction of the winds throughout the year.

Moisture recycling exposure analysis

Dhaka is located in one of the world's largest river deltas, at the confluence of the Ganges, Brahmaputra, and Meghna rivers. However, much of the surface water nearby is heavily polluted, and thus much of the municipal water supply comes from actively re-charged groundwater. Recent efforts to augment household level water supply, by encouraging small-scale rainwater harvesting, are aimed at mitigating reliance on groundwater, though these efforts are very new. In terms of moisture recycling, Dhaka's most important terrestrial sources of precipitation come from the Tibetan plateau, and it is here that key land-use changes would, theoretically, need to be monitored. However, the overwhelming dependence on actively recharged groundwater suggests a significant buffer to surface water change in Dhaka, leading to low exposure to moisture recycling change.

References

1. Kreimer, A, Arnold, M, Carlin, A. Building safer cities. The future of disaster risk. Disaster risk management series, 3. 2003.

2. Andrews, C. Water in Asian Cities: Utilities Performance and Civil Society Views. Asian Development Bank. 2004. Chicago

Dhaka, Bangladesh (*continued*)

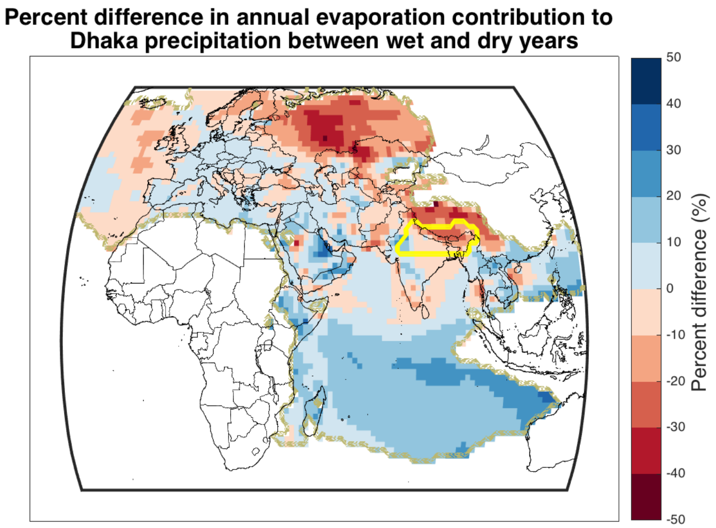

Figure S20: Percent difference in evaporation contribution between driest and wettest years.

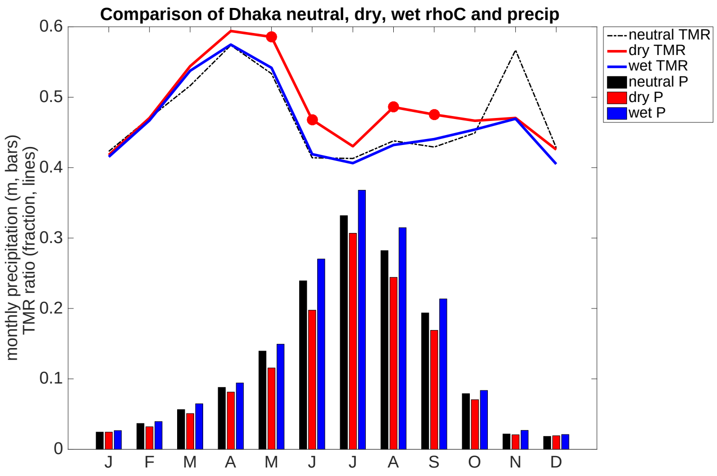

Figure S21: Summary of monthly average precipitation and terrestrial moisture recycling (TMR) during neutral, dry, and wet years. Note that the y-axis corresponds to both m/month of precipitation (represented by bars), and the fraction of precipitation originating from upwind land surfaces (represented by lines). The dots indicate significant differences for either dry or wet years during that month; see Methods in main article for further details.

Guangzhou, China

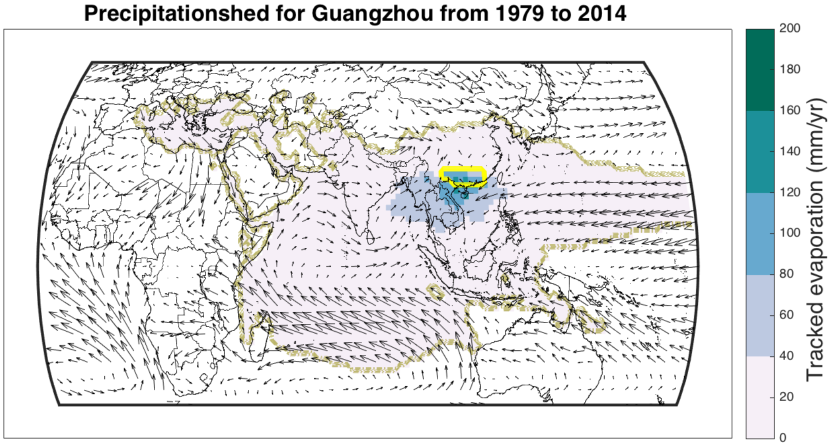

Figure S22: Guangzhou precipitationshed, based on a core boundary (ranging from 1 mm/yr). Yellow lines enclose the sink region, and the prevailing winds are indicated to illustrate the average direction of the winds throughout the year.

Moisture recycling exposure analysis

Guangzhou is one of China's key megacities, and is located in the Pearl River Delta. It receives about 80% of water from surface runoff, which is heavily polluted from agricultural runoff, industrial effluent, and human waste upstream. Gaungzhou receives water from the western (Xi) and Northern (Bei) tributaries, and the primary issue for water security is managing water quality. Although the Pearl River is a large river, the supply of water available is dwindling due to ever growing demands for freshwater both upstream and in the Pearl River Delta. Thus, any changes in moisture recycling are likely to be felt quite quickly given the limited buffers that exist. Thus, we argue that Guangzhou's water security is moderately exposed to moisture recycling dynamics.

References

1. Cosentino-Roush, S, Zhao, W, Zhai, L, Wu, Y. "Ingredients for Protection: How Guanzhou, China can learn from Boston and New York City's Experiences with Surface Drinking Water Source Protection." 2010. Lib.noaa.gov.

2. Ouyang, T, Zhu, Z, Kuang, Y. Assessing impact of urbanization on river water quality in the Pearl River Delta Economic Zone, China. Environmental Monitoring and Assessment. 2006. doi: 10.1007/s10661-005-9064-x

3. Yang, T, Shao, Q, Hao, ZC, Chen, X, Zhang, Z, Xu, CY, et al. Regional frequency analysis and spatio-temporal pattern characterization of rainfall extremes in the Pearl River Basin, China. Journal of Hydrology. 2010. doi: 10.1016/j.jhydrol.2009.11.013

Guangzhou, China (*continued*)

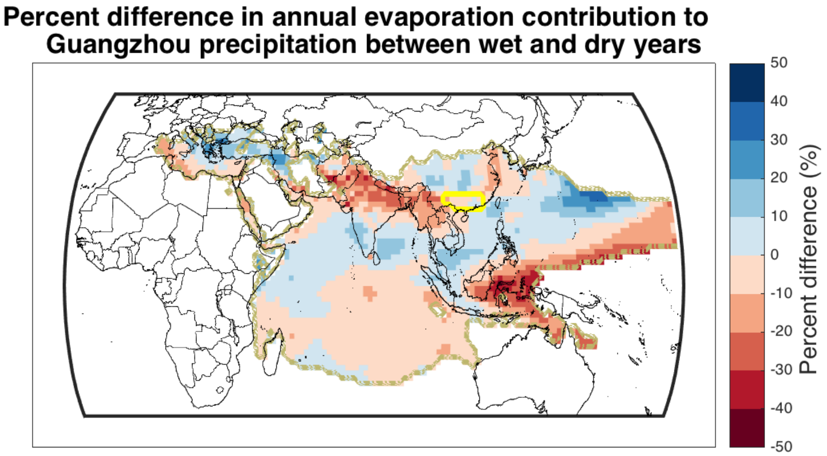

Figure S23: Percent difference in evaporation contribution between driest and wettest years.

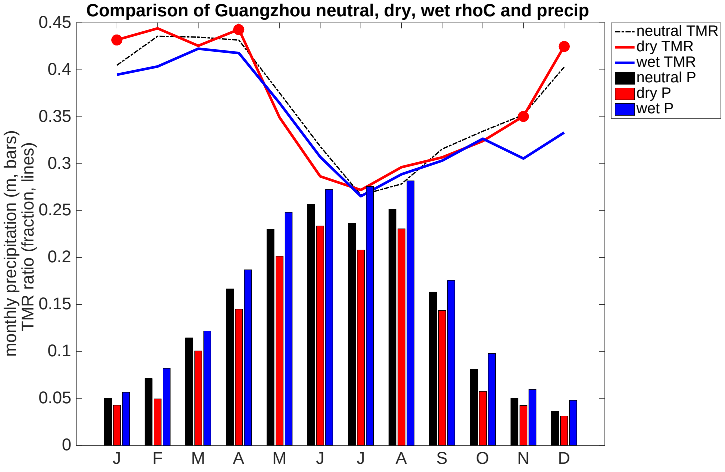

Figure S24: Summary of monthly average precipitation and terrestrial moisture recycling (TMR) during neutral, dry, and wet years. Note that the y-axis corresponds to both m/month of precipitation (represented by bars), and the fraction of precipitation originating from upwind land surfaces (represented by lines). The dots indicate significant differences for either dry or wet years during that month; see Methods in main article for further details.

Istanbul, Turkey

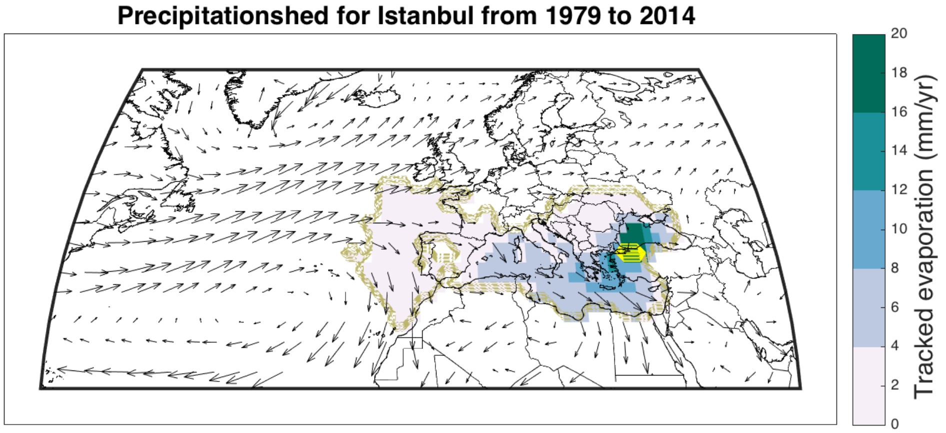

Figure S25: Istanbul precipitationshed, based on a core boundary (ranging from 1 mm/yr). Yellow lines enclose the sink region, and the prevailing winds are indicated to illustrate the average direction of the winds throughout the year.

Moisture recycling exposure analysis

Istanbul is located in a narrow isthmus betwen the Mediterranean Sea and the Black Sea. 97% of water supply comes from two reservoirs systems: the Omerli-Darlik in the east, and the Terkos-Alibeykov in the west. The reservoir networks are located almost entirely within the metropolis, and informal settlements around the reservoirs lead to pollution problems. Planned development of expanded water supply will only just keep pace with growth in demand. Furthermore, several studies have found that Istanbul is sensitive to drought periods. Thus, we consider Istanbul to be highly exposed to changes in moisture recycling.

References

1. Demirci, A, Butt, A. Historical Overview and Current Trends in Istanbul's Water Supply Development, Globalization And Water Resources Management: The Changing Value of Water, August 6-8, Awra/Iwlri-University of Dundee International Specialty Conference. 2001.

2. Gerek, AC, Alp, M, Zuran, A, Sahin, M, Kilinc, I. Present conditions, future potentials, drought analysis and management of reservoirs around Istanbul. In Proceedings of International Congress on River Basin Management (Vol. 1, p. 374e385). 2007.

Istanbul, Turkey (*continued*)

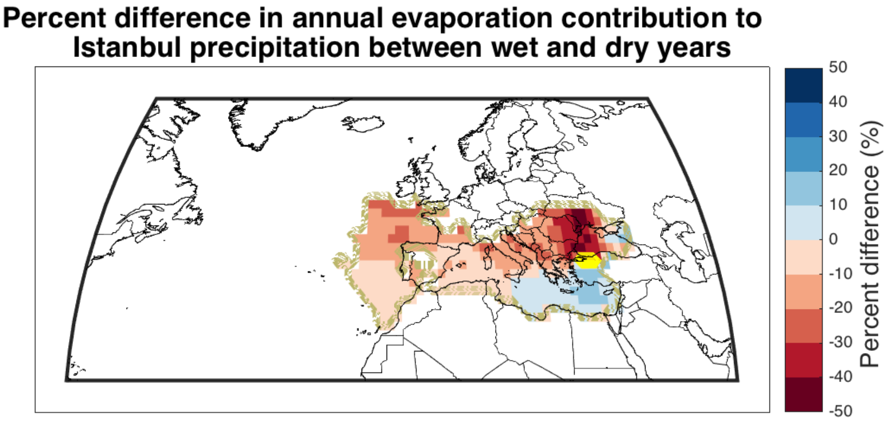

Figure S26: Percent difference in evaporation contribution between driest and wettest years.

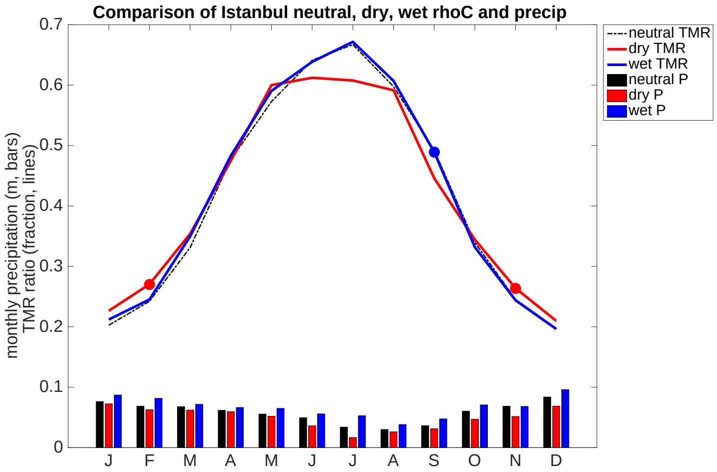

Figure S27: Summary of monthly average precipitation and terrestrial moisture recycling (TMR) during neutral, dry, and wet years. Note that the y-axis corresponds to both m/month of precipitation (represented by bars), and the fraction of precipitation originating from upwind land surfaces (represented by lines). The dots indicate significant differences for either dry or wet years during that month; see Methods in main article for further details.

Jakarta, Indonesia

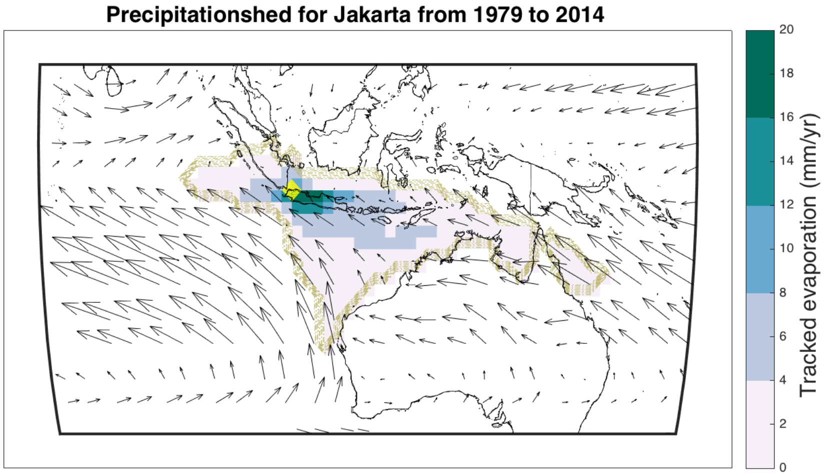

Figure S28: Jakarta precipitationshed, based on a core boundary (ranging from 1 mm/yr). Yellow lines enclose the sink region, and the prevailing winds are indicated to illustrate the average direction of the winds throughout the year.

Moisture recycling exposure analysis

Jakarta receives most of its municipal water (80%) from surface runoff from the Citarum river to the east, transported via the West Tarum canal. The water supplied to the city is distributed unevenly with large gaps in access between the rich and the poor. The water supply seems to be stable and the biggest issue is improving infrastructure and piped connections. Though moisture recycling may be affected by ongoing land-use change, the dominance of oceanic sources of moisture (versus land sources) suggests that Jakarta has low exposure to moisture recycling dynamics.

References

1. Fulazzaky, MA. Water quality evaluation system to assess the status and the suitability of the Citarum river water to different uses. Environmental monitoring and assessment. 2010. 168(1), 669-684.

2. Asian Development Bank. Report and Recommendation of the President to the Board of Directors: Proposed Loan to the Republic of Indonesia: West Jakarta Water Supply Development Project. 2007.

Jakarta, Indonesia (*continued*)

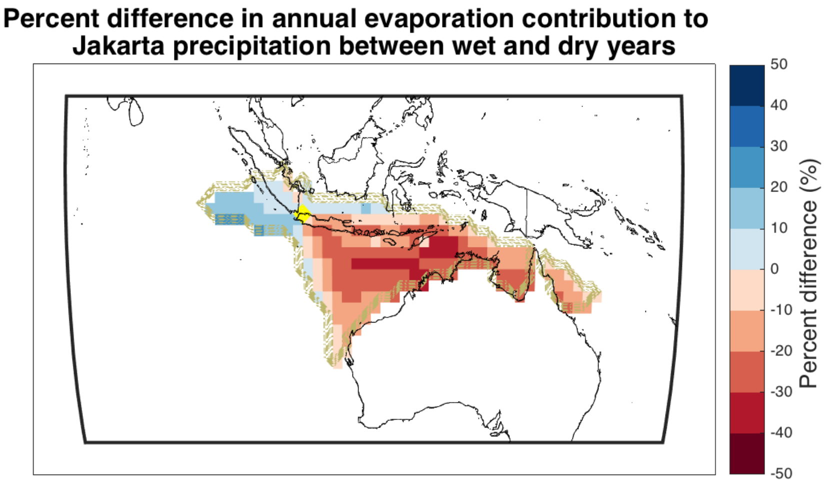

Figure S29: Percent difference in evaporation contribution between driest and wettest years.

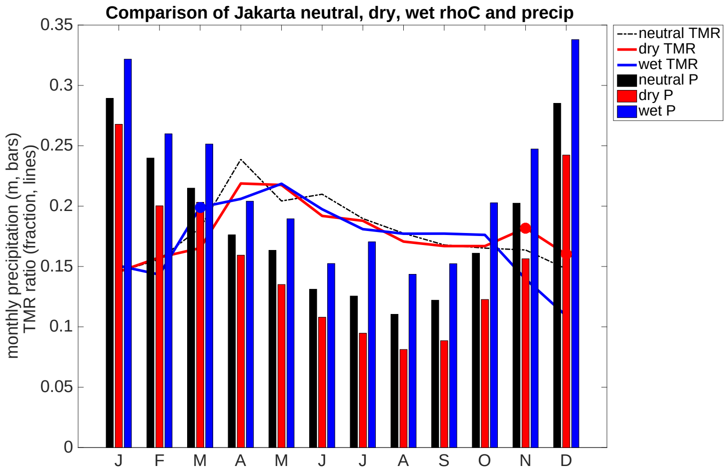

Figure S30: Summary of monthly average precipitation and terrestrial moisture recycling (TMR) during neutral, dry, and wet years. Note that the y-axis corresponds to both m/month of precipitation (represented by bars), and the fraction of precipitation originating from upwind land surfaces (represented by lines). The dots indicate significant differences for either dry or wet years during that month; see Methods in main article for further details.

Kolkata, India

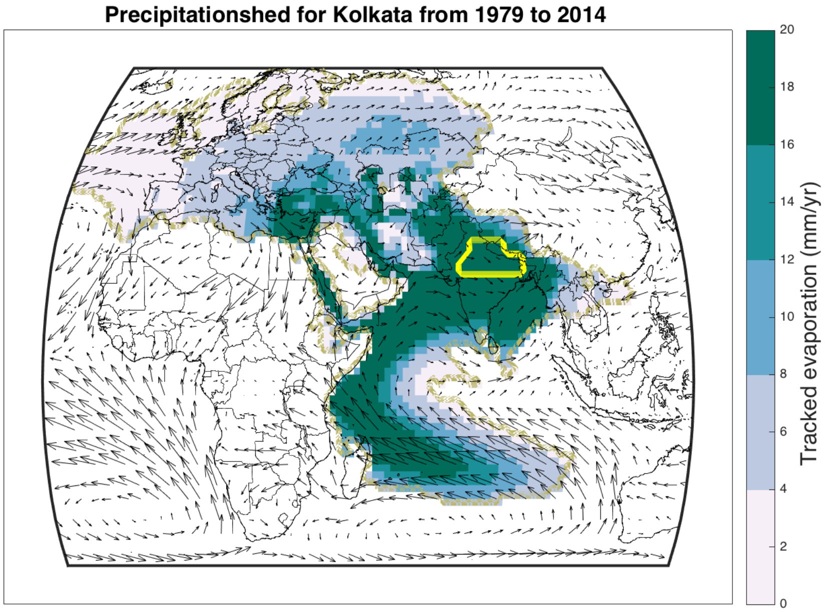

Figure S31: Kolkata precipitationshed, based on a core boundary (ranging from 1 mm/yr). Yellow lines enclose the sink region, and the prevailing winds are indicated to illustrate the average direction of the winds throughout the year.

Moisture recycling exposure analysis

Kolkata receives most of its water from the Hooghly River, a distributary of the Ganges River. There is relatively reliable flow and modern infrastructure enables considerably better water availability than in many other Indian cities. Flooding is one of the key water-related problems in Kolkata, and combined with the large size of the basin, the blended snowmelt and runoff system, the exposure to moisture recycling dynamics and water security is considered low.

References

1. Ray, B, Shaw, R. Water Stress in the Megacity of Kolkata, India, and Its Implications for Urban Resilience. Urban Disasters and Resilience in Asia. 2016 (317)

2. Juran, L, Lahiri-Dutt, K. Waterscapes in transition: changing uses and perceptions of water in middle class homes in Kolkata, India. Water History. 2017. 1-19.

Kolkata, India (*continued*)

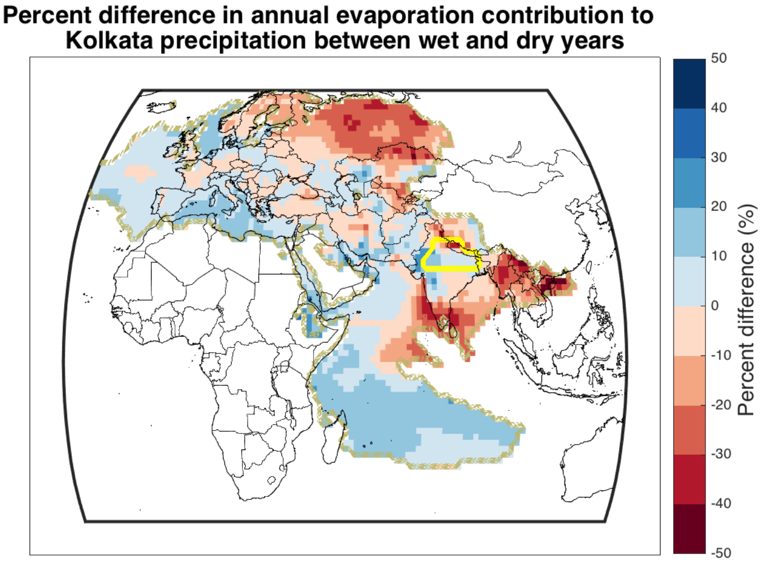

Figure S32: Percent difference in evaporation contribution between driest and wettest years.

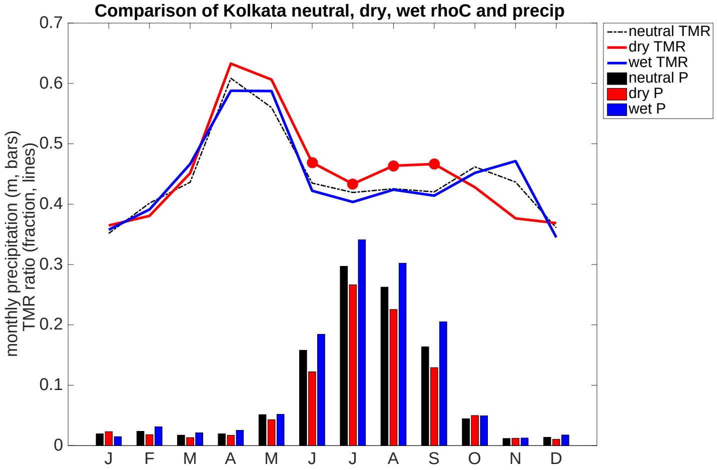

Figure S33: Summary of monthly average precipitation and terrestrial moisture recycling (TMR) during neutral, dry, and wet years. Note that the y-axis corresponds to both m/month of precipitation (represented by bars), and the fraction of precipitation originating from upwind land surfaces (represented by lines). The dots indicate significant differences for either dry or wet years during that month; see Methods in main article for further details.

Lagos, Nigeria

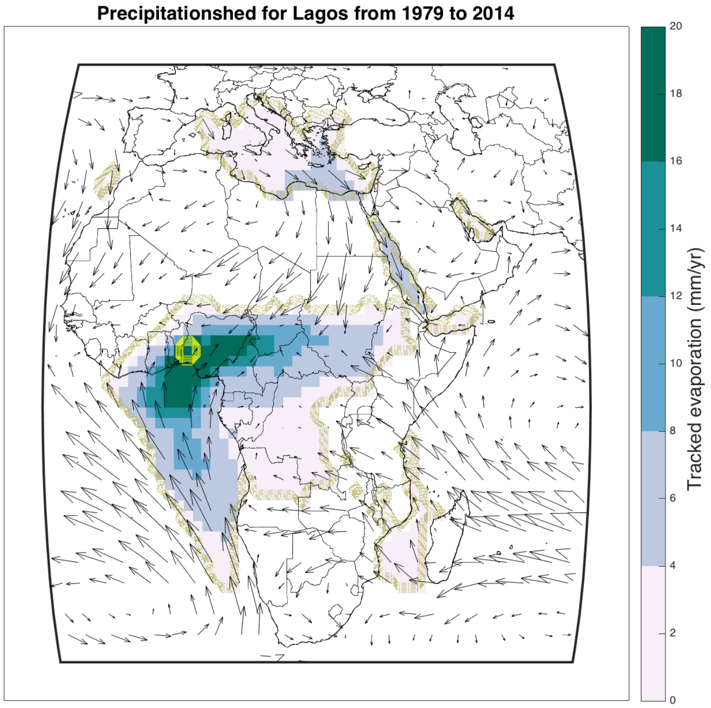

Figure S34: Lagos precipitationshed, based on a core boundary (ranging from 1 mm/yr). Yellow lines enclose the sink region, and the prevailing winds are indicated to illustrate the average direction of the winds throughout the year.

Moisture recycling exposure analysis

Lagos is a coastal city that relies on surface runoff from the Ogun River (in addition to several other smaller rivers). Recent analyses suggest that only 10% of Lagos residents receive piped water, suggesting an enormous infrastructure and management challenge. However, there is considerable rainfall of terrestrial origin, and there is little to no reservoir storage to provide a buffer. Thus, given the immediacy of impact from changes in rainfall felt in the population, Lagos is considered highly exposed to moisture recycling dynamics.

References

1. Jideonwo, JA. Ensuring sustainable water supply in Lagos, Nigeria. Masters Thesis. University of Pennsylvania. 2014

2. Omole, DO, Ndambuki, JM, Badejo, AA, Oyewo, DO, Soyemi, TO. Public feedback on State of domestic water supply in Lagos: Implications for public health. Indian Journal of Traditional Knowledge. 2016. 15(2), 245-253.

3. Owolabi, A. Increasing population, urbanization and climatic factors in Lagos State, Nigeria: The nexus and implications on water demand and supply. Journal of Global Initiatives: Policy, Pedagogy, Perspective. 2017. 11(2), 6.

Lagos, Nigeria (*continued*)

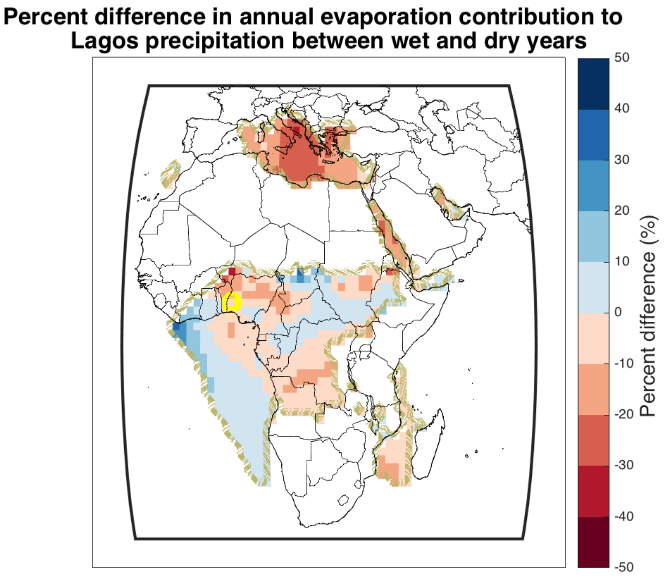

Figure S35: Percent difference in evaporation contribution between driest and wettest years.

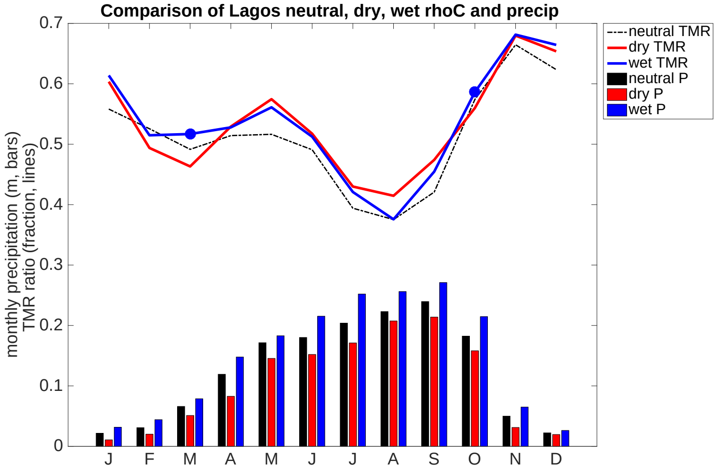

Figure S36: Summary of monthly average precipitation and terrestrial moisture recycling (TMR) during neutral, dry, and wet years. Note that the y-axis corresponds to both m/month of precipitation (represented by bars), and the fraction of precipitation originating from upwind land surfaces (represented by lines). The dots indicate significant differences for either dry or wet years during that month; see Methods in main article for further details.

Los Angeles, USA

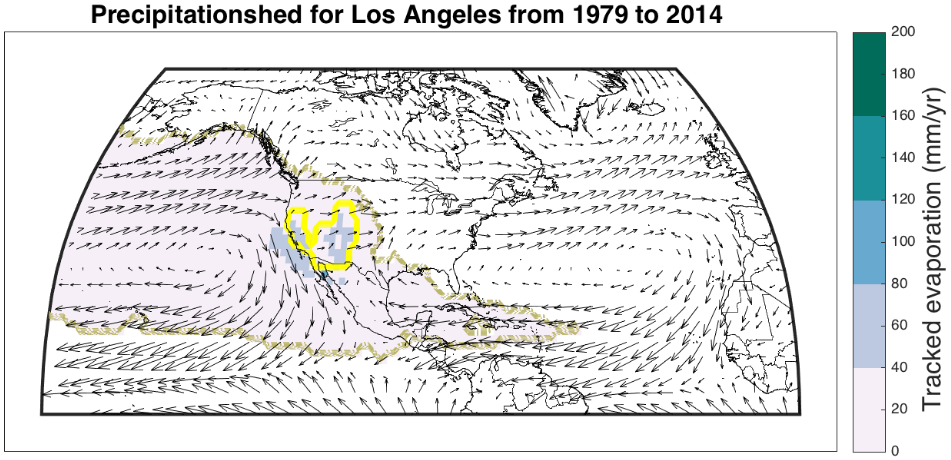

Figure S37: Los Angeles precipitationshed, based on a core boundary (ranging from 1 mm/yr). Yellow lines enclose the sink region, and the prevailing winds are indicated to illustrate the average direction of the winds throughout the year.

Moisture recycling exposure analysis

Los Angeles, unlike many megacities, receives water from numerous watersheds across the southwestern United States, including the Colorado River, the Owens River, and the San Joaquin-Sacramento River. All of these water resources are fully- or over-allocated. Much of the Californian water passes through both desert and agricultural land, while the Colorado River is snowmelt passing through the harshest deserts in the United States. Despite the arid climate and the over-allocation, there is some amount of buffer within the system to re-allocate water from agriculture to municipal uses, and the prospect of moisture recycling change impacting Los Angeles' water resources appears moderate, so we consider Los Angeles to be moderately exposed to moisture recycling dynamics.

References

1. Pagán, BR, Ashfaq, M, Rastogi, D, Kendall, DR, Kao, SC, Naz, BS et al. Extreme hydrological changes in the southwestern US drive reductions in water supply to Southern California by mid century. Environmental Research Letters. 2016. doi: 10.1088/1748-9326/11/9/094026

2. State Water Resources Control Board - Los Angeles. 2017. Waterboards.ca.gov.

3. LA County Waterworks Districts — Your Water — Water Sources . 2017. Dpw.lacounty.gov.

Los Angeles, USA (*continued*)

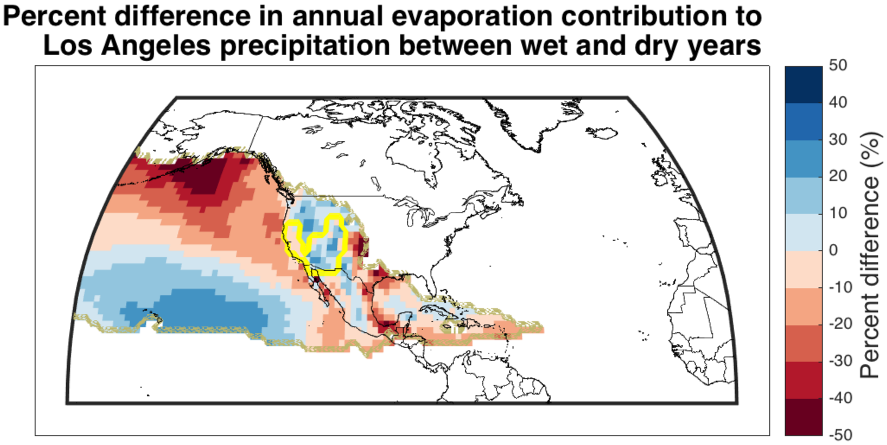

Figure S38: Percent difference in evaporation contribution between driest and wettest years.

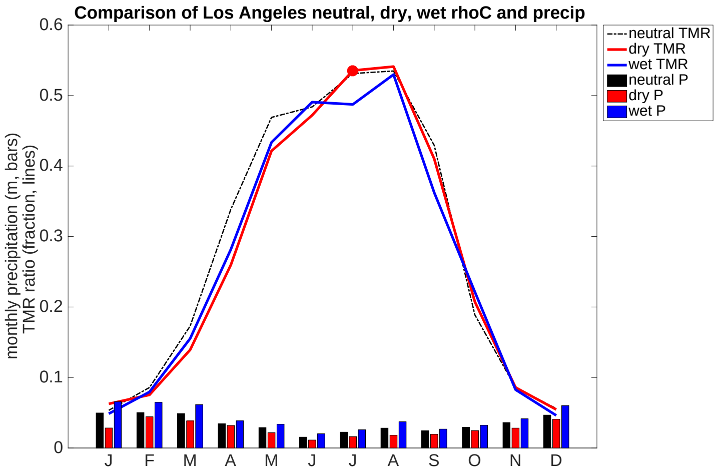

Figure S39: Summary of monthly average precipitation and terrestrial moisture recycling (TMR) during neutral, dry, and wet years. Note that the y-axis corresponds to both m/month of precipitation (represented by bars), and the fraction of precipitation originating from upwind land surfaces (represented by lines). The dots indicate significant differences for either dry or wet years during that month; see Methods in main article for further details.

Manila, Philippines

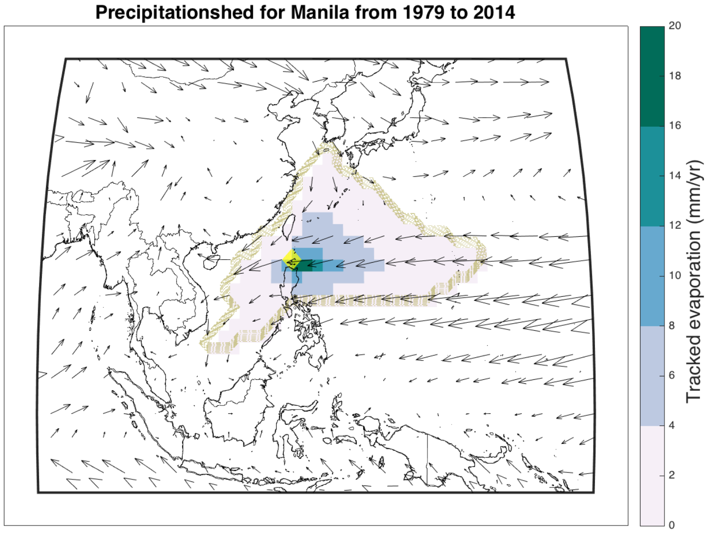

Figure S40: Manila precipitationshed, based on a core boundary (ranging from 1 mm/yr). Yellow lines enclose the sink region, and the prevailing winds are indicated to illustrate the average direction of the winds throughout the year.

Moisture recycling exposure analysis

Manila is served by the Angat-Ipo-La Mesa water system, with the Angat Dam providing about 90% of the water for Metro Manila. The watershed for the reservoir is relatively small, but highly protected providing good source water protection. The importance of moisture recycling is relatively low, with internal moisture recycling being nearly negligible. Given the well-protected design of the water supply and the low importance of terrestrial moisture recycling, Manila's exposure to moisture recycling change is considered low.

References

1. Shah, SH, Zerriffi, H. Urban water demand, climatic variation, and irrigation-water insecurity: interactive stressors and lessons for water governance from the Angat River basin (Philippines). Water International. 2017. doi: 10.1080/02508060.2017.1342073

2. Manila Water Company, Inc. — Home . 2017. Manilawater.com.

3. Provincial Government of Bulacan Online. 2017. Bulacan.gov.ph.

Manila, Philippines (*continued*)

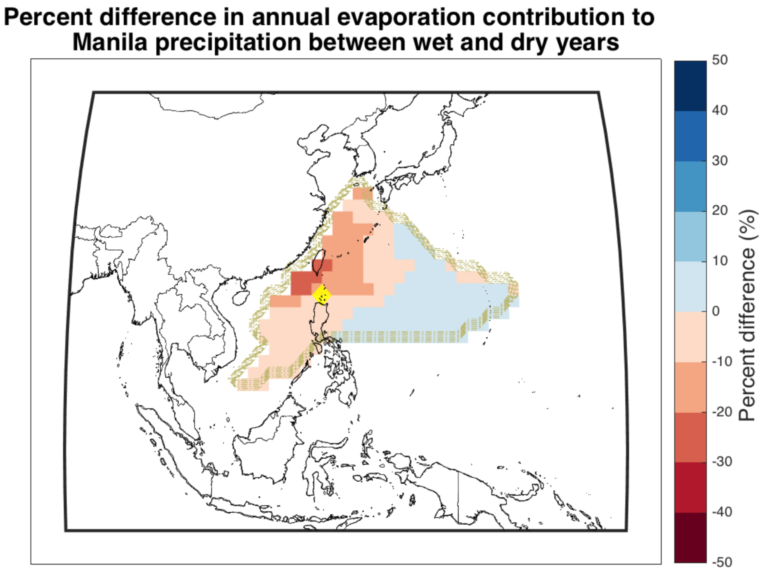

Figure S41: Percent difference in evaporation contribution between driest and wettest years.

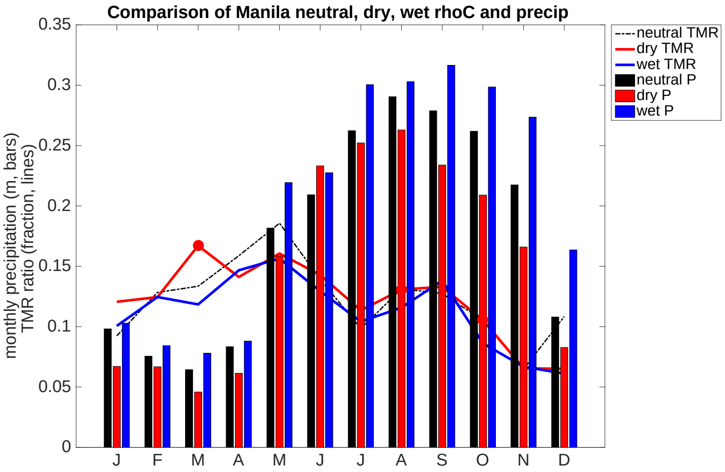

Figure S42: Summary of monthly average precipitation and terrestrial moisture recycling (TMR) during neutral, dry, and wet years. Note that the y-axis corresponds to both m/month of precipitation (represented by bars), and the fraction of precipitation originating from upwind land surfaces (represented by lines). The dots indicate significant differences for either dry or wet years during that month; see Methods in main article for further details.

Mexico City, Mexico

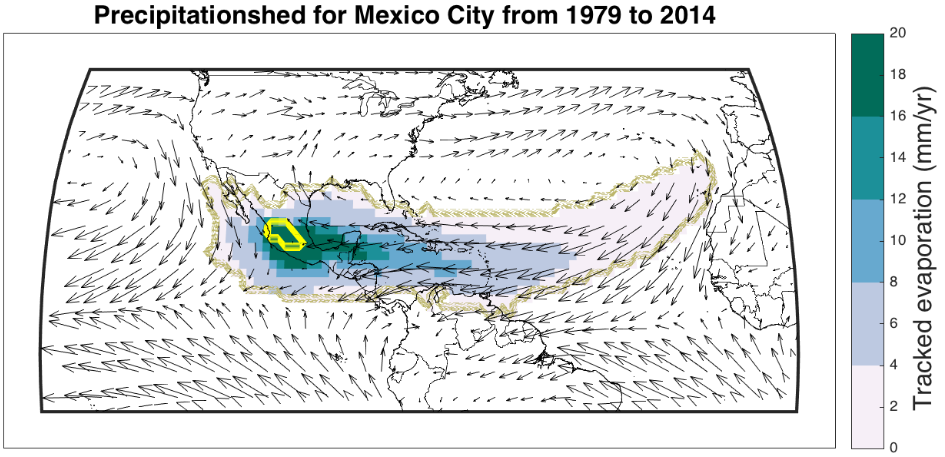

Figure S43: Mexico City precipitationshed, based on a core boundary (ranging from 1 mm/yr). Yellow lines enclose the sink region, and the prevailing winds are indicated to illustrate the average direction of the winds throughout the year.

Moisture recycling exposure analysis

Mexico City is primarily reliant on actively recharged groundwater supplies including private wells, supplemented by about 20% from the nearby Cutzamala River. The rapid growth of Mexico City has taxed groundwater supplies, leading to over-exploitation, land subsidence, and falling groundwater tables. Mexico City is semi-arid, and is rapidly growing, and will be increasingly reliant on surface water resources (though from where it is unclear). If groundwater is channeled through centralized systems and water supply networks, moisture recycling exposure would by low. However, given that the population is in direct access of the water cycle (via distributed, actively recharged groundwater wells), and that terrestrial moisture recycling is relatively high (30%), we suggest that Mexico City is moderately exposed to moisture recycling dynamics.

References

1. Spring, ÚO. Aquatic systems and water security in the Metropolitan Valley of Mexico City. Current Opinion in Environmental Sustainability. 2011. doi: 10.1016/j.cosust.2011.11.002

2. Martnez, PF, Bandala, ER. Issues and challenges for water supply, storm water drainage and wastewater treatment in the Mexico City metropolitan area. Water and Cities in Latin America: Challenges for Sustainable Development. Routeledge. 2015 109-125.

3. Spring, ÚO. Managing water resources in Mexico in the context of climate change. In Managing Water Resources under Climate Uncertainty (pp. 377-403). Springer International Publishing. 2015.

Mexico City, Mexico (*continued*)

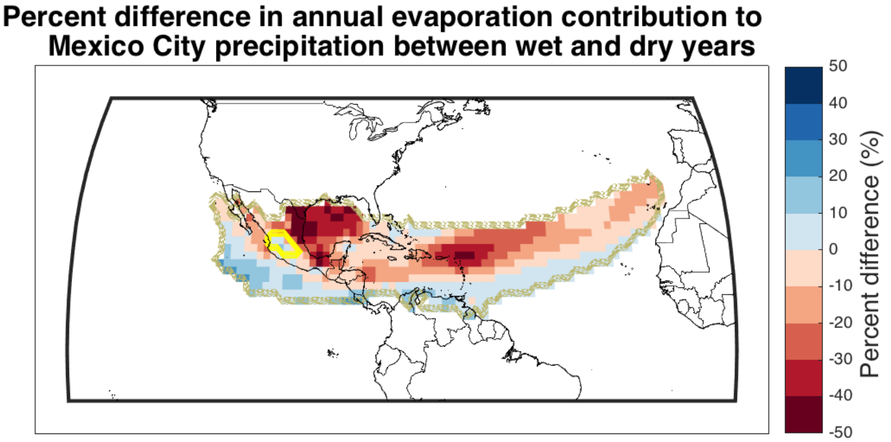

Figure S44: Percent difference in evaporation contribution between driest and wettest years.

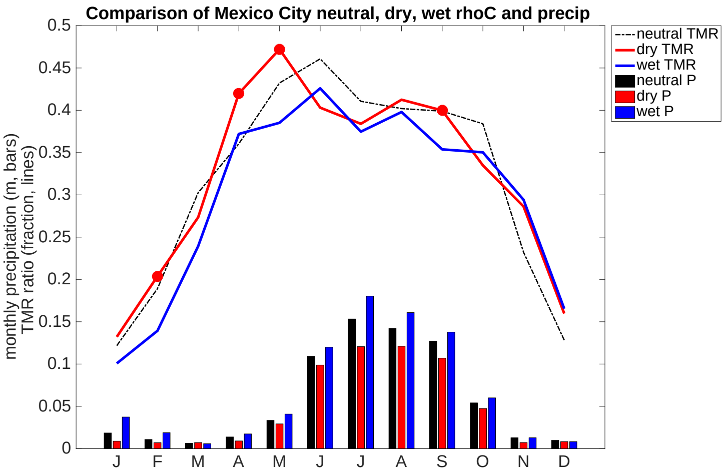

Figure S45: Summary of monthly average precipitation and terrestrial moisture recycling (TMR) during neutral, dry, and wet years. Note that the y-axis corresponds to both m/month of precipitation (represented by bars), and the fraction of precipitation originating from upwind land surfaces (represented by lines). The dots indicate significant differences for either dry or wet years during that month; see Methods in main article for further details.

Moscow, Russia

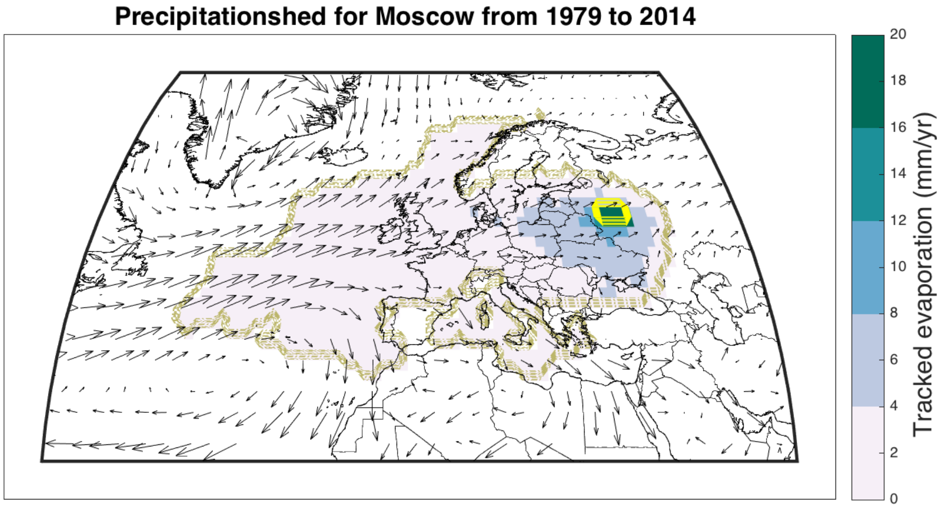

Figure S46: Moscow precipitationshed, based on a core boundary (ranging from 1 mm/yr). Yellow lines enclose the sink region, and the prevailing winds are indicated to illustrate the average direction of the winds throughout the year.

Moisture recycling exposure analysis

Moscow receives most of its freshwater from the Ivankovo Reservoir on the upper reaches of the Volga River. There are no other major sources of water supply to Moscow. Both terrestrial moisture recycling and vegetation-regulated moisture recycling are high. Historically, landscapes in the Upper Volga have been quite sensitive to dry and wet years, leading to extensive forest loss and fires. Furthermore, there is a high potential for land-use change (anthropogenic or natural) from e.g. agricultural change and forest fire. All of these conditions suggest Moscow is highly exposed to changes in moisture recycling.

References

1. Panyushkina, IP, Karpukhin, AA, Engovatova, AV. Moisture record of the Upper Volga catchment between AD 1430 and 1600 supported by a δ-13 C tree-ring chronology of archaeological pine timbers. Dendrochronologia. 2016. 39, 24-31.

2. Rai, IN, Shoba, S, Shchegolkova, N, Dzhamalov, R, Venitsianov, E, Santosa, IGN, et al. Analysis of the specifics of water resources management in regions with rapidly growing population under different climate conditions: Case study of Bali Island and the Moscow Region. Water resources. 2015. 42(5), 735-746.

Moscow, Russia (*continued*)

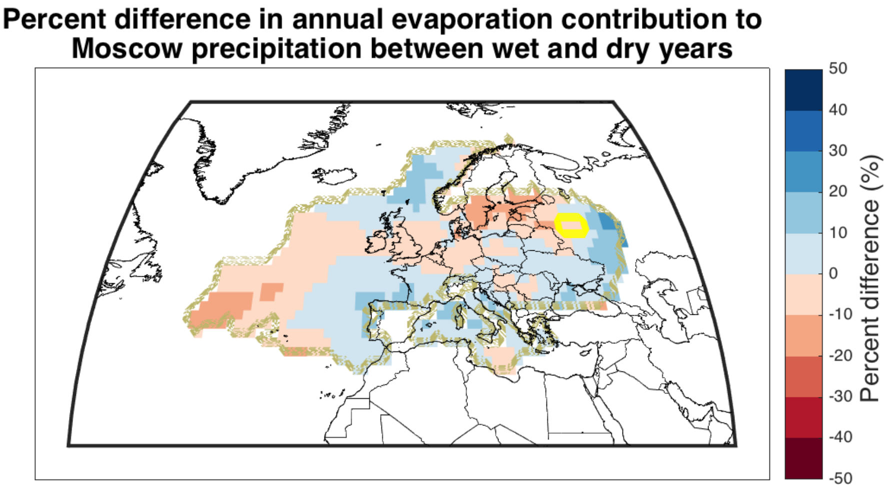

Figure S47: Percent difference in evaporation contribution between driest and wettest years.

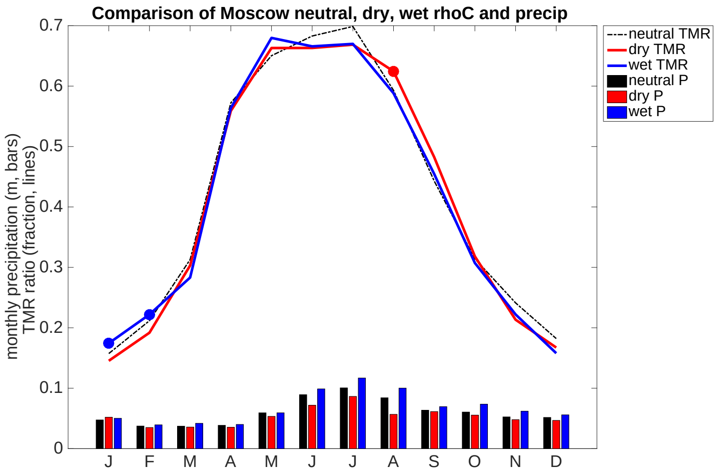

Figure S48: Summary of monthly average precipitation and terrestrial moisture recycling (TMR) during neutral, dry, and wet years. Note that the y-axis corresponds to both m/month of precipitation (represented by bars), and the fraction of precipitation originating from upwind land surfaces (represented by lines). The dots indicate significant differences for either dry or wet years during that month; see Methods in main article for further details.

Mumbai, India

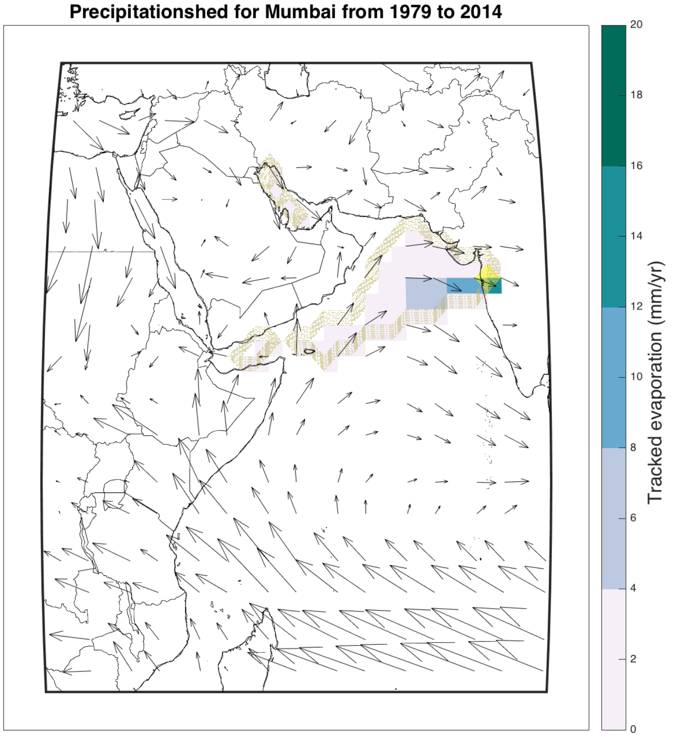

Figure S49: Mumbai precipitationshed, based on a core boundary (ranging from 1 mm/yr). Yellow lines enclose the sink region, and the prevailing winds are indicated to illustrate the average direction of the winds throughout the year.

Moisture recycling exposure analysis

Mumbai receives its water from several rivers running off the Western Ghat Mountains, and is primarily stored in the Bhandup reservoir complex. The importance of moisture recycling for this rainfall is relatively high, and though the runoff is supplied by Monsoon rains, dry seasons can be particularly risky leaving reservoirs empty. We consider Mumbai to be highly exposed to moisture recycling dynamics.

References

1. Mumbai City. Mumbai City Development Plan 2005-2025.

2. Singh, S, Mishra, A. Deforestation-induced costs on the drinking water supplies of the Mumbai metropolitan, India. Global environmental change. 2014. doi: 10.1016/j.gloenvcha.2014.04.0[illegible]

3. Holthaus, E.. India's Monsoon "the Most Important Weather Phenomenon in the World" Is Way Behind Schedule. Slate Magazine. 2014.

Mumbai, India (*continued*)

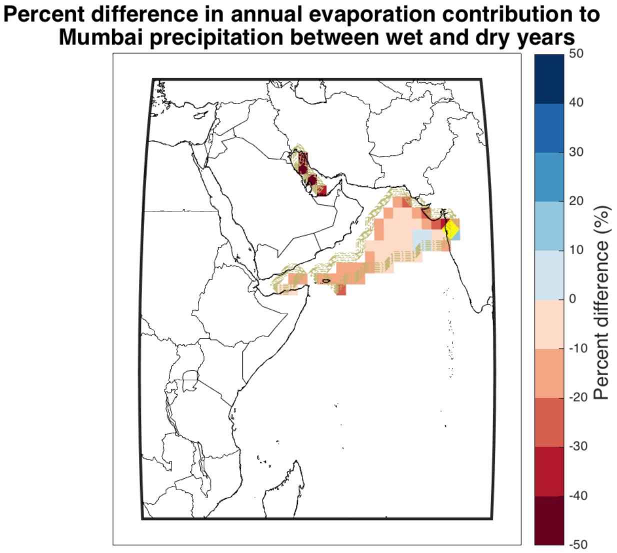

Figure S50: Percent difference in evaporation contribution between driest and wettest years.

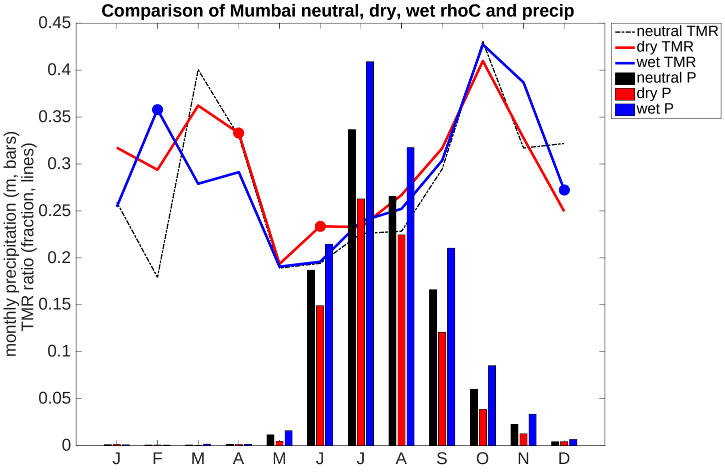

Figure S51: Summary of monthly average precipitation and terrestrial moisture recycling (TMR) during neutral, dry, and wet years. Note that the y-axis corresponds to both m/month of precipitation (represented by bars), and the fraction of precipitation originating from upwind land surfaces (represented by lines). The dots indicate significant differences for either dry or wet years during that month; see Methods in main article for further details.

New York City, USA

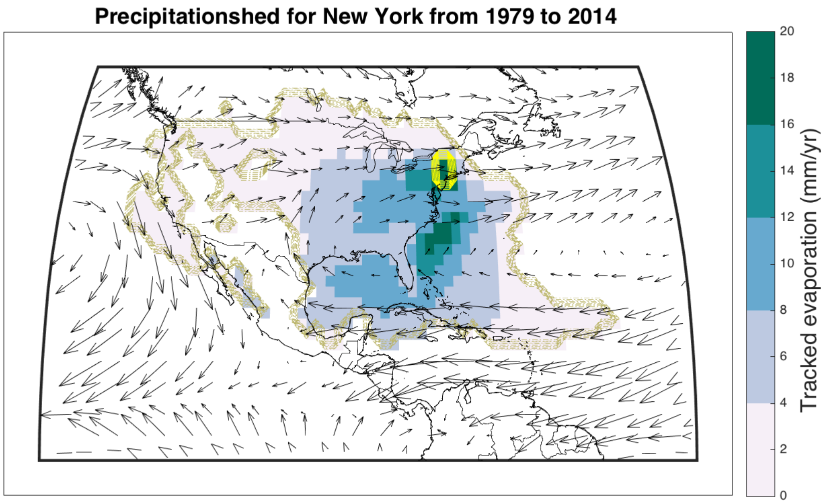

Figure S52: New York City precipitationshed, based on a core boundary (ranging from 1 mm/yr). Yellow lines enclose the sink region, and the prevailing winds are indicated to illustrate the average direction of the winds throughout the year.

Moisture recycling exposure analysis

New York City receives 90% of its water from the Catskill Mountains via the Catskill Aqueduct and the Delaware Aqueduct. The watersheds are very well protected from development and pollution, providing excellent benefits in terms of water quality. The city has experienced drought somewhat recently leading to mandatory water conservation efforts. Given this drought sensitivity, the reliance on surface runoff from one region, and the relatively high moisture recycling values, we consider New York City to be highly exposed to its moisture recycling dynamics .

References

1. Hanlon, JW. Complementary safeguards for robust regional watershed governance in a federation: New York City and its municipal water supply. Environmental Science & Policy. 2017. doi: 10.1016/j.envsci.2017.04.014

2. New York City EPA. Drought Watch Declared For New York City Water Supply. 2001.

3. Merguerian, C. NYMC talk on Geology of the NYC Aqueduct System. Talk given to the Long Island Geologists Dinner Meeting. 2010.

New York City, USA (*continued*)

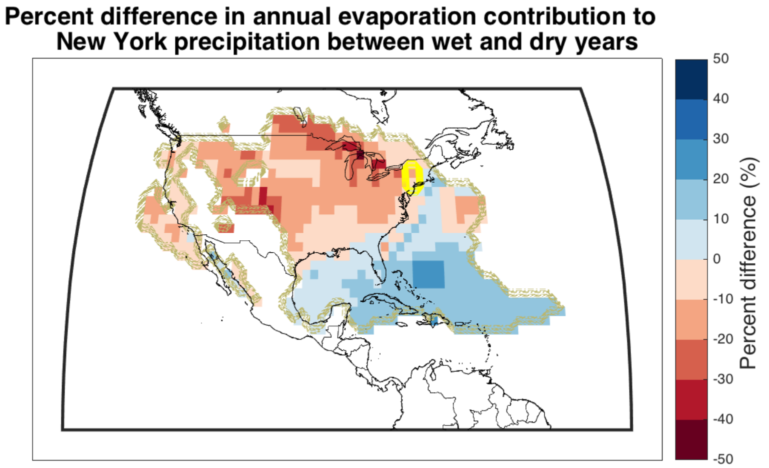

Figure S53: Percent difference in evaporation contribution between driest and wettest years.

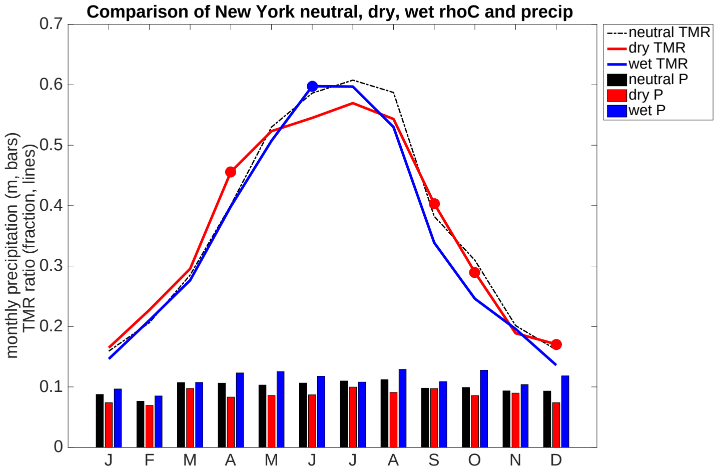

Figure S54: Summary of monthly average precipitation and terrestrial moisture recycling (TMR) during neutral, dry, and wet years. Note that the y-axis corresponds to both m/month of precipitation (represented by bars), and the fraction of precipitation originating from upwind land surfaces (represented by lines). The dots indicate significant differences for either dry or wet years during that month; see Methods in main article for further details.

Osaka-Kobe, Japan

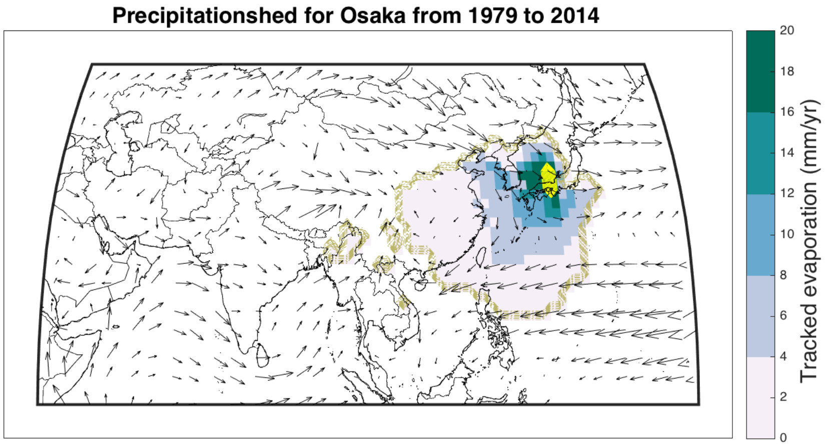

Figure S55: Osaka-Kobe precipitationshed, based on a core boundary (ranging from 1 mm/yr). Yellow lines enclose the sink region, and the prevailing winds are indicated to illustrate the average direction of the winds throughout the year.

Moisture recycling exposure analysis

The Osaka-Kobe metropolitan area receives its water from the Kyu-Yodo River, flowing out of Lake Biwa. This river supplies water to many other downstream users, though streamflow seems to be less sensitive to drought, and water management is state-of-the-art and efficient. Given the reliance on surface runoff, the moderate reliance on terrestrial evaporation, and the buffers in water storage in the water management system, we consider Osaka-Kobe to be moderately exposed to moisture recycling dynamics.

References

1. Kumagai, M, Vincent, WF, Ishikawa, K, Aota, Y. Lessons from Lake Biwa and other Asian Lakes: Global and local perspectives. In Freshwater management (pp. 1-22). Springer Japan. 2003.

2. GESAP. Water Supply System in Osaka, Japan. 2017.

Osaka-Kobe, Japan (*continued*)

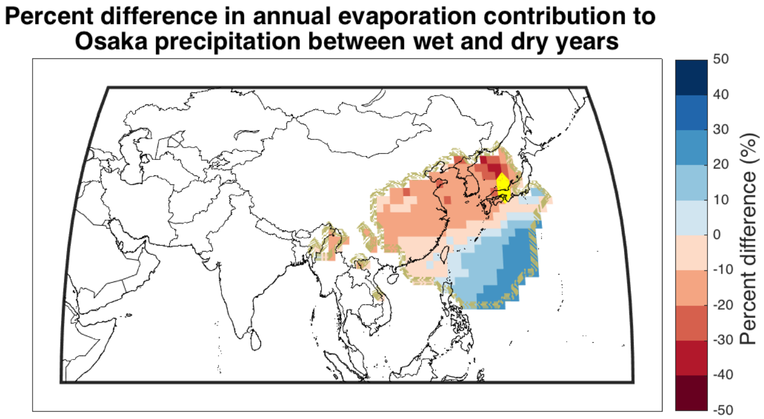

Figure S56: Percent difference in evaporation contribution between driest and wettest years.

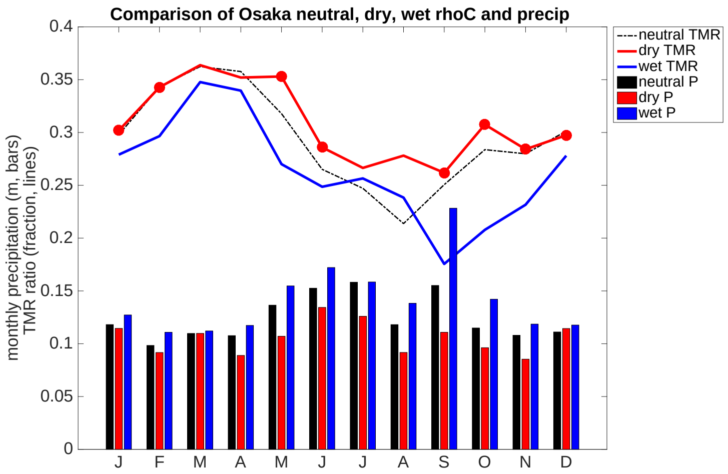

Figure S57: Summary of monthly average precipitation and terrestrial moisture recycling (TMR) during neutral, dry, and wet years. Note that the y-axis corresponds to both m/month of precipitation (represented by bars), and the fraction of precipitation originating from upwind land surfaces (represented by lines). The dots indicate significant differences for either dry or wet years during that month; see Methods in main article for further details.

Paris, France

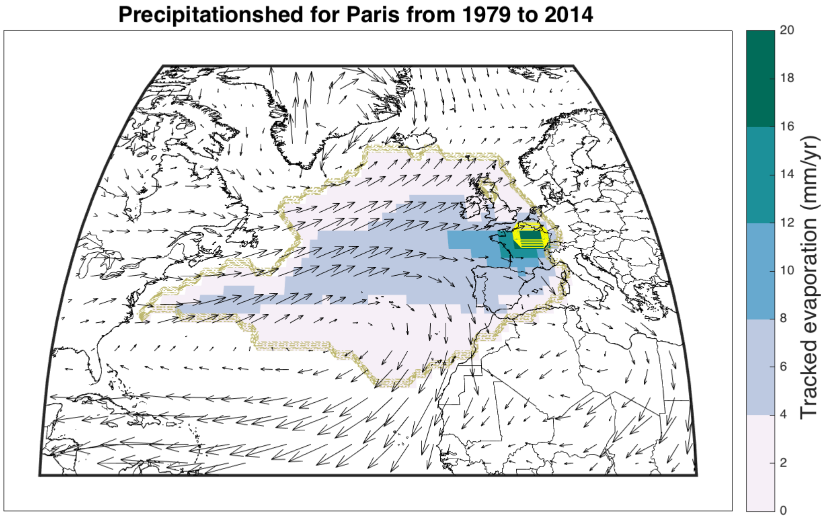

Figure S58: Paris precipitationshed, based on a core boundary (ranging from 1 mm/yr). Yellow lines enclose the sink region, and the prevailing winds are indicated to illustrate the average direction of the winds throughout the year.

Moisture recycling exposure analysis

Paris receives its water from a dense network of surface and groundwater supplies, transported via tunnel and aqueduct and stored in both above- and below-ground reservoirs. Moisture recycling rates are relatively high, with significant recycling occurring within France itself. The distributed nature of the supply points, however, does not necessarily reduce the overall connection to moisture recycling, so we consider Paris' water security is moderately exposed to moisture recycling.

References

1. Loubet, P, Roux, P, Guérin-Schneider, L, Bellon-Maurel, V. Life cycle assessment of forecasting scenarios for urban water management: A first implementation of the WaLA model on Paris suburban area. Water research. 2016. doi:10.1016/j.watres.2015.12.008

2. Dorchies, D, Thirel, G, Perrin, C, Bader, JC, Thepot, R, Rizzoli, JL, et al. Climate change impacts on water resources and reservoir management in the Seine river basin (France). La Houille Blanche. doi:10.1051/lhb/2016047

3. le Strat, A. The remunicipalization of Paris' water supply service. [presentation]

Paris, France (*continued*)

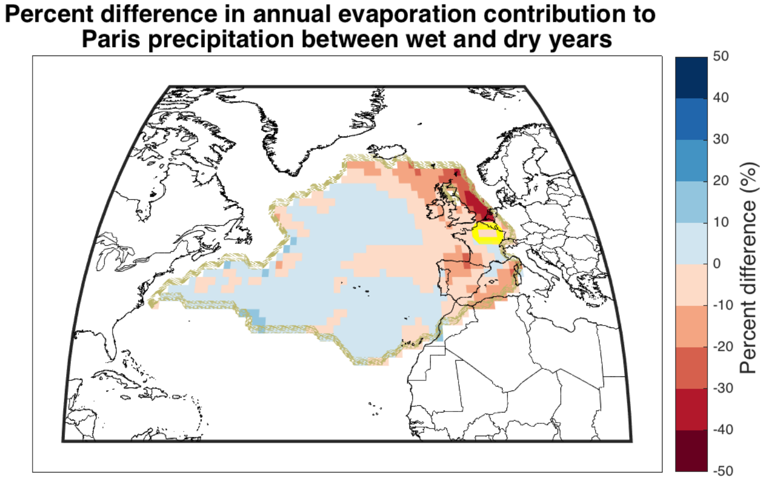

Figure S59: Percent difference in evaporation contribution between driest and wettest years.

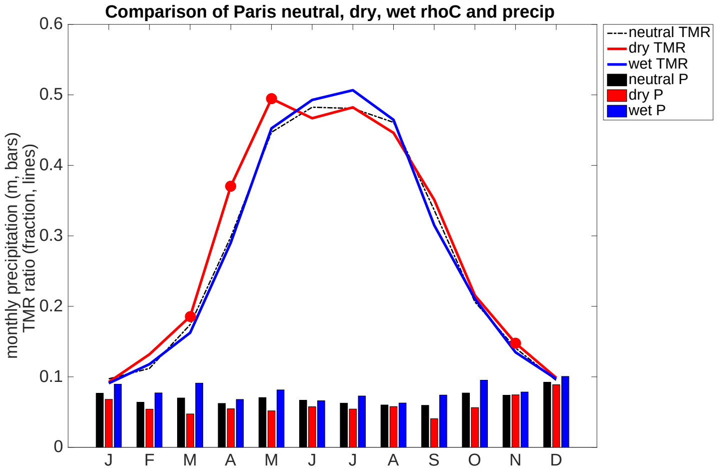

Figure S60: Summary of monthly average precipitation and terrestrial moisture recycling (TMR) during neutral, dry, and wet years. Note that the y-axis corresponds to both m/month of precipitation (represented by bars), and the fraction of precipitation originating from upwind land surfaces (represented by lines). The dots indicate significant differences for either dry or wet years during that month; see Methods in main article for further details.

Rio de Janeiro, Brazil

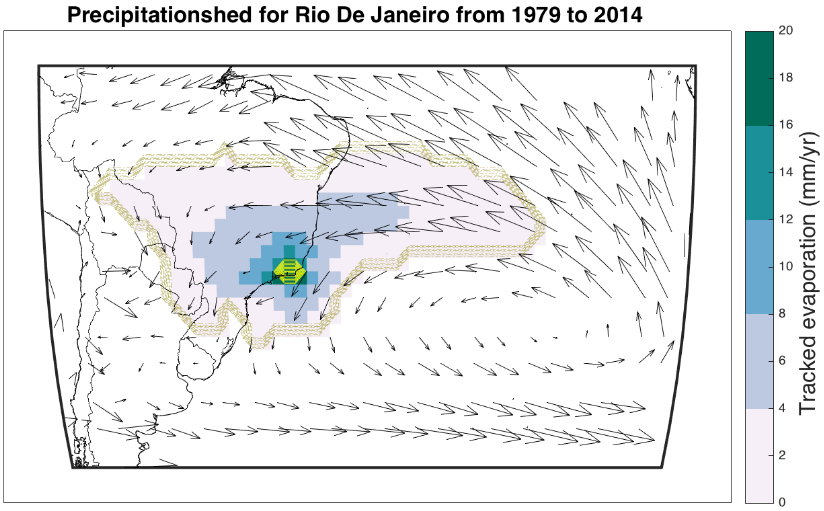

Figure S61: Rio de Janeiro precipitationshed, based on a core boundary (ranging from 1 mm/yr). Yellow lines enclose the sink region, and the prevailing winds are indicated to illustrate the average direction of the winds throughout the year.

Moisture recycling exposure analysis

Rio de Janeiro receives most of its drinking water from the Guandu River and a portion of the Paraiba do Sul River. This surface runoff is generated locally, and is in a region with very high (and growing) water demand. Moisture recycling is relatively high, as is vegetation-regulation of this moisture recycling, but it occurs primarily outside of the watershed. Given the reliance on surface runoff, the high moisture recycling ratios, and the vulnerability of water security to drought, we consider Rio de Janeiro to be highly exposed to moisture recycling dynamics.

References

1. Formiga-Johnsson, RM. Water Resources Management in Brazil: Challenges and New Perspectives. World Bank Water. 2014.

2. Britto, AL, Formiga-Johnsson, RM, Carneiro, PRF. Water supply and hydrosocial scarcity in the Rio de Janeiro Metropolitan Area. Ambiente & Sociedade. 2016. 19(1), 183-206.

Rio de Janeiro, Brazil (*continued*)

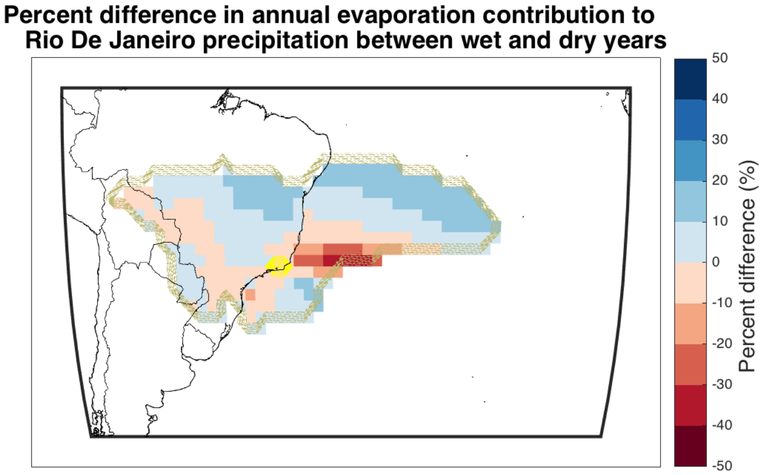

Figure S62: Percent difference in evaporation contribution between driest and wettest years.

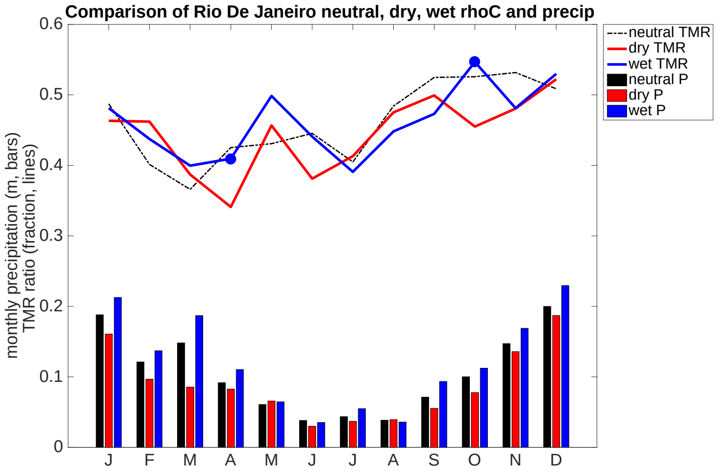

Figure S63: Summary of monthly average precipitation and terrestrial moisture recycling (TMR) during neutral, dry, and wet years. Note that the y-axis corresponds to both m/month of precipitation (represented by bars), and the fraction of precipitation originating from upwind land surfaces (represented by lines). The dots indicate significant differences for either dry or wet years during that month; see Methods in main article for further details.

Shanghai, China

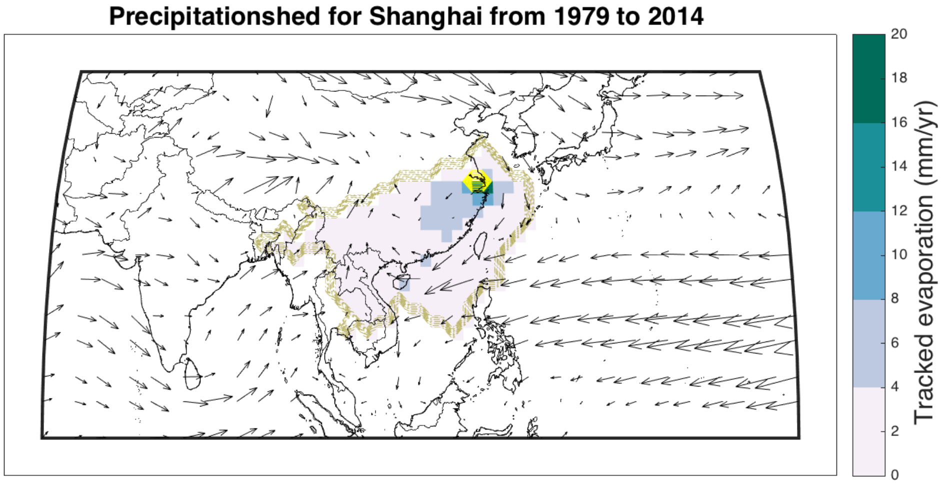

Figure S64: Shanghai precipitationshed, based on a core boundary (ranging from 1 mm/yr). Yellow lines enclose the sink region, and the prevailing winds are indicated to illustrate the average direction of the winds throughout the year.

Moisture recycling exposure analysis

Shanghai draws its drinking water from the Huangpu river, the final tributary of the Yangtze before it empties into the East China Sea. The basin is very small, and is subject to a great deal of pollution from the metropolitan area of Shanghai, as well as agriculture further upstream. Terrestrial moisture recycling is quite high, and is also sensitive to drier than normal conditions. Given the low availability of additional surface water resources, the importance of moisture recycling, and sensitivity to dry conditions, we consider Shanghai's water security to be highly exposed to moisture recycling dynamics.

References

1. Helmer, R, Hespanhol, I. Case Study II* - Shanghai Huangpu River, China. Water Pollution Control - A Guide to the Use of Water Quality Management Principles. WHO/UNEP. 1997.

2. Wang, X, Wang, S, Peng, G, Katz, D, Ling, H. Ecological restoration for river ecosystems: comparing the Huangpu River in Shanghai and the Hudson River in New York. Ecosystem Health and Sustainability. 2015. 1(7), 1-14.

Shanghai, China (*continued*)

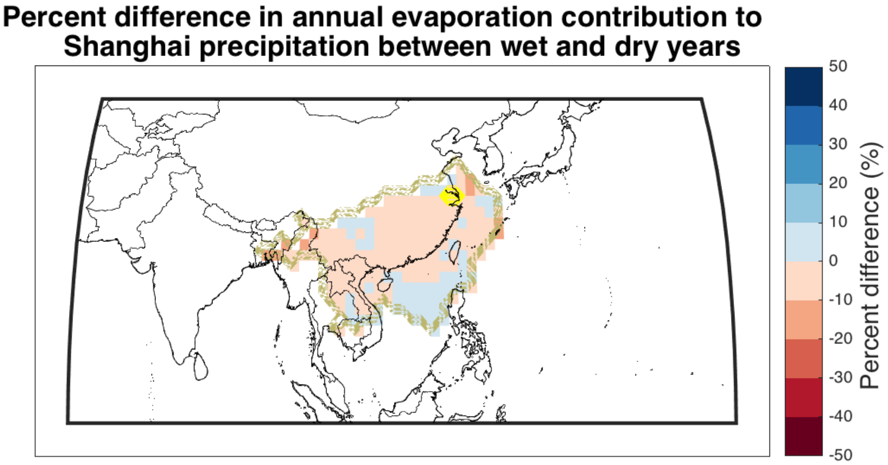

Figure S65: Percent difference in evaporation contribution between driest and wettest years.

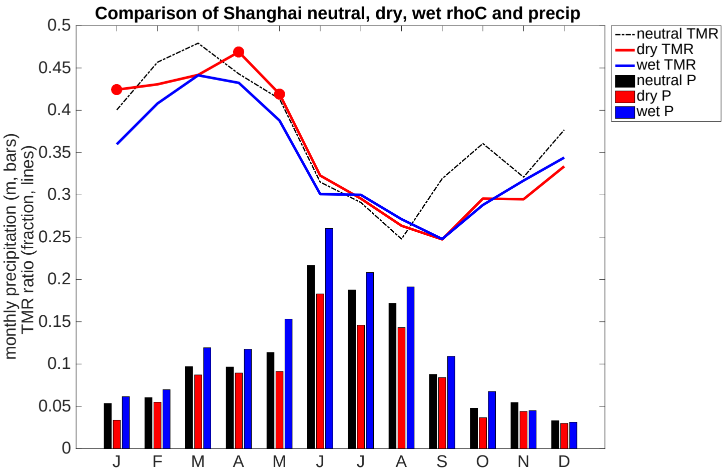

Figure S66: Summary of monthly average precipitation and terrestrial moisture recycling (TMR) during neutral, dry, and wet years. Note that the y-axis corresponds to both m/month of precipitation (represented by bars), and the fraction of precipitation originating from upwind land surfaces (represented by lines). The dots indicate significant differences for either dry or wet years during that month; see Methods in main article for further details.

Shenzhen, China

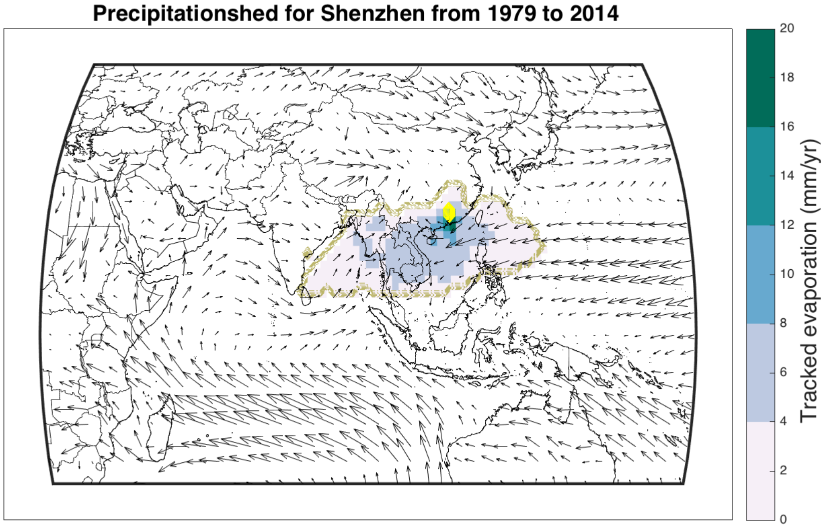

Figure S67: Shenzhen precipitationshed, based on a core boundary (ranging from 1 mm/yr). Yellow lines enclose the sink region, and the prevailing winds are indicated to illustrate the average direction of the winds throughout the year.

Moisture recycling exposure analysis

Shenzhen receives its water from the eastern tributary of the Pearl River (aka Dongjiang). It is a small catchment located to the northeast of the Pearl River Delta. It provides water to both Hong Kong and Shenzhen (as well as smaller settlements). The river is fully allocated and any variation in supply will be felt immediately by the users of water. Thus, any change in moisture recycling dynamics would be felt very quickly by the users of the Dongjiang, and we suggest that Shenzhen's water security is highly exposed to moisture recycling dynamics.

References

1. Zhang, R, Chen, J, Cui, G. Drought and Water Supply Reliability of East River (Dongjiang) Basin in South China. Advances in Water Resources and Hydraulic Engineering. 2009. 296-300.

2. Liu, C. One Chinese City's Struggle With Water Scarcity. The Diplomat. 2017.

3. Liu, S. Dongjiang overloaded. Chinadialogue.net. 2012.

4. Kao, E. A turning tide that could leave Hong Kong in deep water. South China Morning Post. 2015.

Shenzhen, China (*continued*)

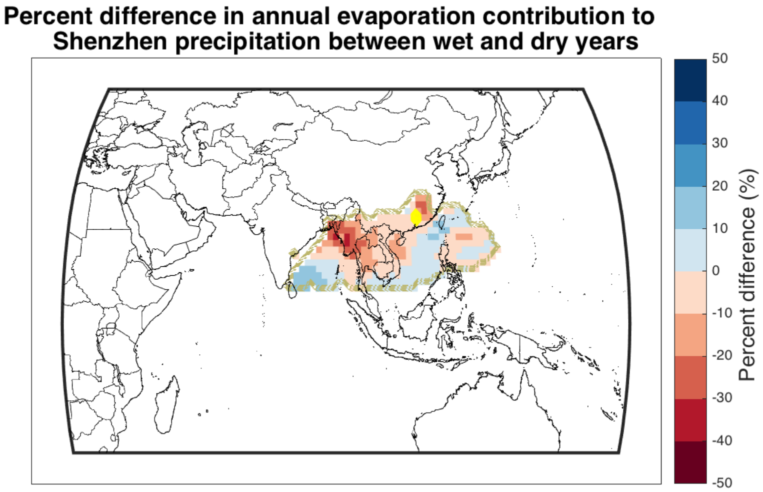

Figure S68: Percent difference in evaporation contribution between driest and wettest years.

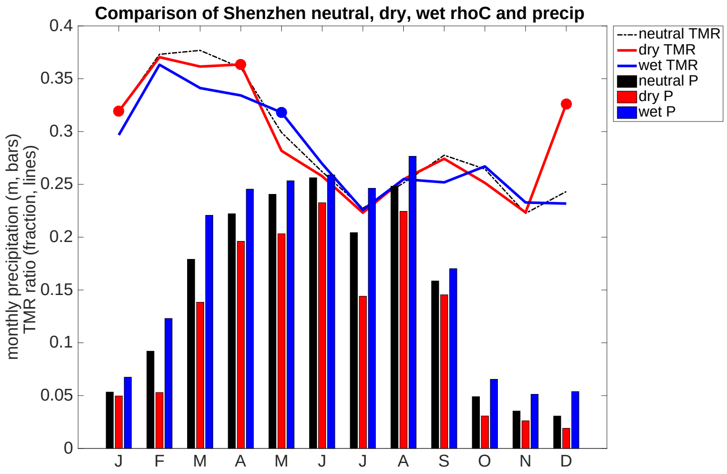

Figure S69: Summary of monthly average precipitation and terrestrial moisture recycling (TMR) during neutral, dry, and wet years. Note that the y-axis corresponds to both m/month of precipitation (represented by bars), and the fraction of precipitation originating from upwind land surfaces (represented by lines). The dots indicate significant differences for either dry or wet years during that month; see Methods in main article for further details.

Tokyo, Japan

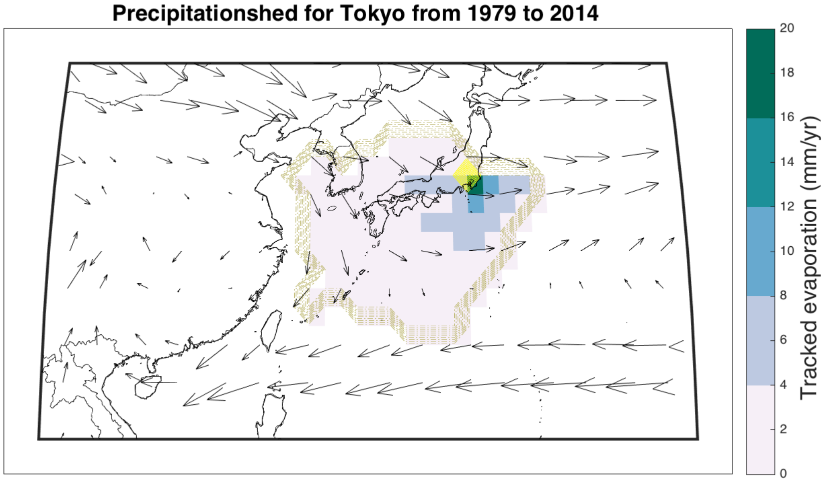

Figure S70: Tokyo precipitationshed, based on a core boundary (ranging from 1 mm/yr). Yellow lines enclose the sink region, and the prevailing winds are indicated to illustrate the average direction of the winds throughout the year.

Moisture recycling exposure analysis

Tokyo draws most of its water from the Arakawa River (78%) and Tama River (19%), with well-established conservation forests surrounding the watersheds to protect surface water quality. The interaction of forests and the watershed appear to be an important component to water security in Tokyo, and moisture recycling is relatively high, with vegetation supporting more than 10% of rainfall in the basin. Given the limited scope of expanded supply from other sources, we consider Tokyo's water security to be moderately exposed to its moisture recycling.

References

1. Bureau of Waterworks Tokyo Metropolitan Government. Water Supply in Tokyo.

2. Hayashi, T, Tokunaga, T, Aichi, M, Shimada, J, Taniguchi, M. Effects of human activities and urbanization on groundwater environments: an example from the aquifer system of Tokyo and the surrounding area. Science of the total environment. 2009. doi: 10.1016/j.scitotenv.2008.07.012

Tokyo, Japan (*continued*)

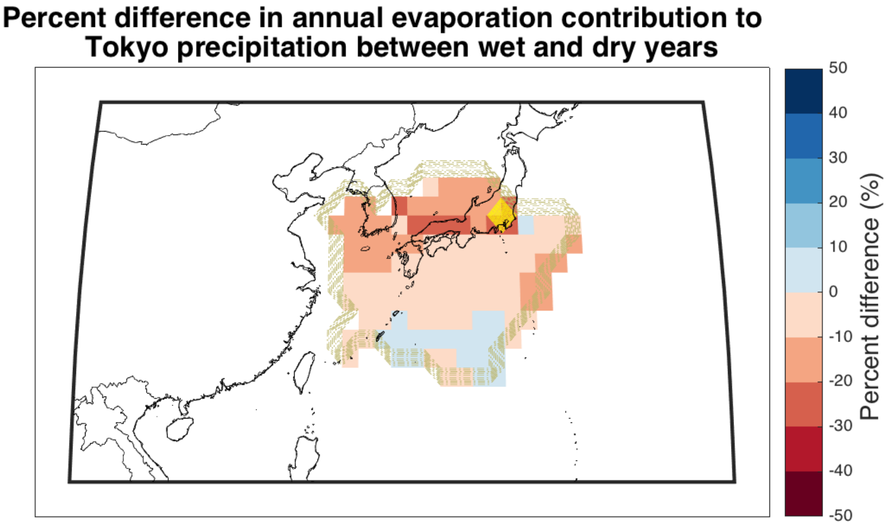

Figure S71: Percent difference in evaporation contribution between driest and wettest years.

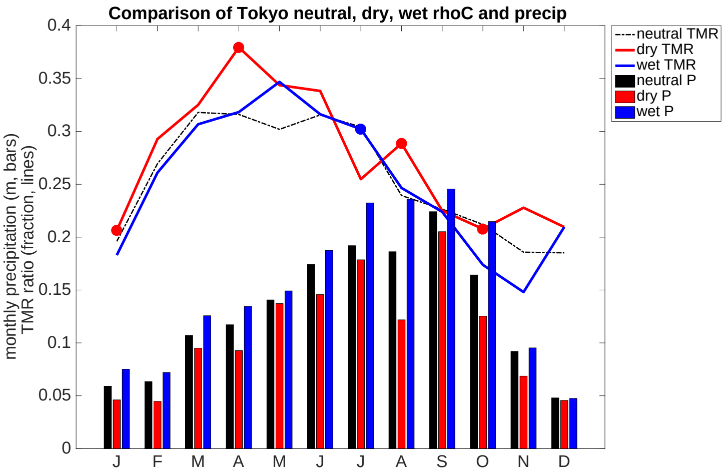

Figure S72: Summary of monthly average precipitation and terrestrial moisture recycling (TMR) during neutral, dry, and wet years. Note that the y-axis corresponds to both m/month of precipitation (represented by bars), and the fraction of precipitation originating from upwind land surfaces (represented by lines). The dots indicate significant differences for either dry or wet years during that month; see Methods in main article for further details.

Wuhan, China

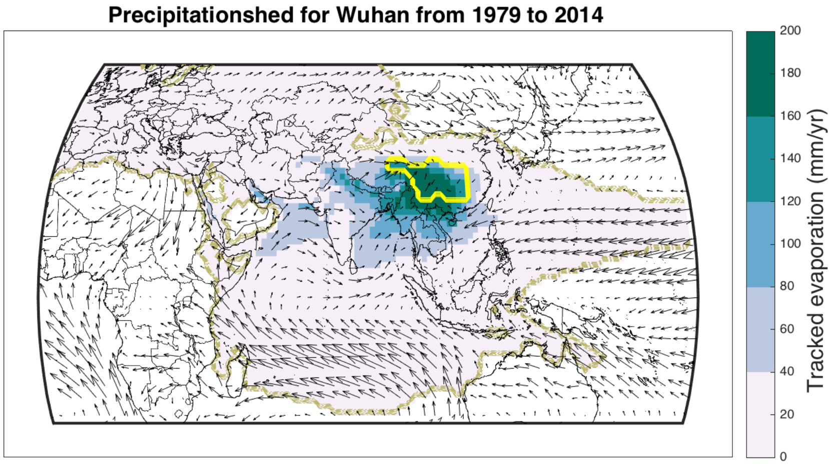

Figure S73: Wuhan precipitationshed, based on a core boundary (ranging from 1 mm/yr). Yellow lines enclose the sink region, and the prevailing winds are indicated to illustrate the average direction of the winds throughout the year.

Moisture recycling exposure analysis

Wuhan draws some of its water from the Hanjiang river, a tributary of the Yangtze, as well as from the Yangtze river proper. In recent years the Yangtze has experienced significant pollution from agriculture and industry leading to large-scale water supply shut-offs within the city. The basin experiences high moisture recycling, with more than 30% of moisture regulated by vegetation. Given the lack of additional water supply sources, due to overwhelming pollution, and the importance of moisture recycling, we consider Wuhan to be highly exposed to changes in moisture recycling.

References

1. Lu, X, Lu, Y, Yang, X, Dong, X, Ma, K, Xiao, S, et al. Mutagenicity of drinking water sampled from the Yangtze River and Hanshui River (Wuhan section) and correlations with water quality parameters. Scientific reports, 2015. 5, 9572.

2. China's Wuhan Hit by Drinking Water Pollution Scare. Radio Free Asia. 2017.

Wuhan, China (*continued*)

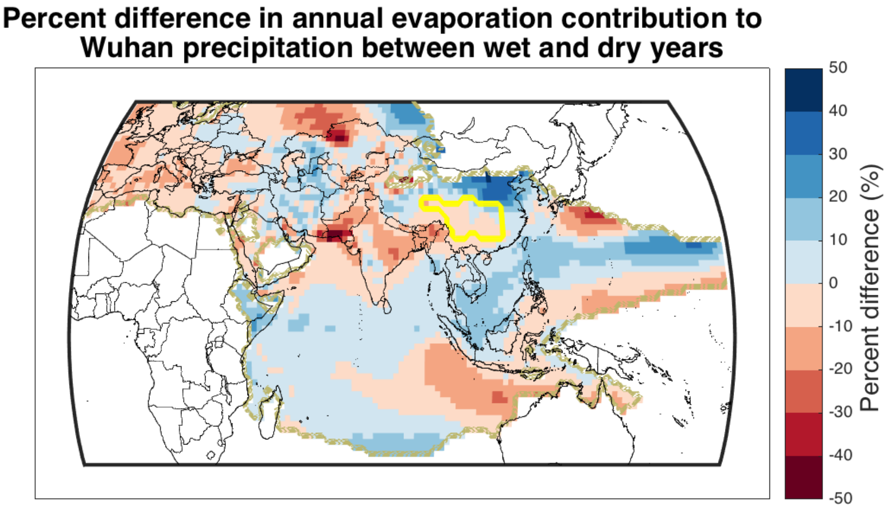

Figure S74: Percent difference in evaporation contribution between driest and wettest years.

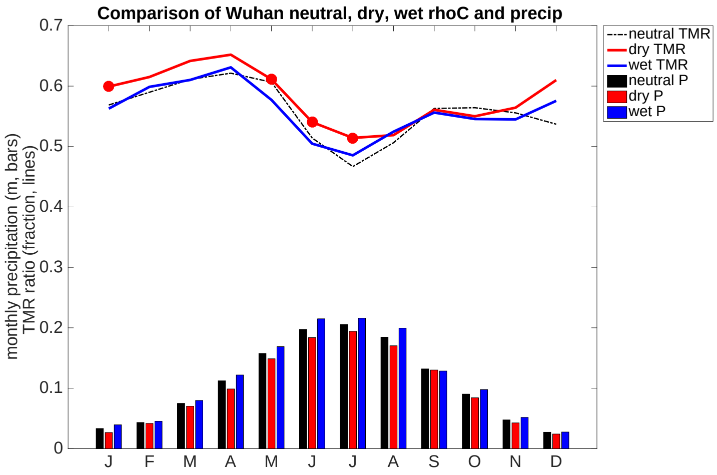

Figure S75: Summary of monthly average precipitation and terrestrial moisture recycling (TMR) during neutral, dry, and wet years. Note that the y-axis corresponds to both m/month of precipitation (represented by bars), and the fraction of precipitation originating from upwind land surfaces (represented by lines). The dots indicate significant differences for either dry or wet years during that month; see Methods in main article for further details.
